# Identification of a second glycoform of the clinically prevalent O1 antigen from *Klebsiella pneumoniae*

**DOI:** 10.1073/pnas.2301302120

**Published:** 2023-07-10

**Authors:** Steven D. Kelly, Olga G. Ovchinnikova, Fabian Müller, Michael Steffen, Martin Braun, Ryan P. Sweeney, Michael Kowarik, Rainer Follador, Todd L. Lowary, Fabio Serventi, Chris Whitfield

**Affiliations:** ^a^Department of Molecular and Cellular Biology, University of Guelph, Guelph, ON N1G 2W1, Canada; ^b^LimmaTech Biologics AG, Schlieren 8952, Switzerland; ^c^Department of Chemistry, University of Alberta, Edmonton, AB T6G 2G2, Canada; ^d^Institute of Biological Chemistry, Academia Sinica, Taipei, Nangang 11529, Taiwan; ^e^Institute of Biochemical Sciences, National Taiwan University, Taipei 10617, Taiwan

**Keywords:** *Klebsiella pneumoniae*, lipopolysaccharide, O antigen, vaccine candidate, antigenic diversity

## Abstract

Carbapenemase and extended β-lactamase-producing *Klebsiella pneumoniae* isolates represent a major health threat, stimulating increasing interest in immunotherapeutic approaches for combating *Klebsiella* infections. Lipopolysaccharide O antigen polysaccharides offer viable targets for immunotherapeutic development, and several studies have described protection with O-specific antibodies in animal models of infection. O1 antigen is produced by almost half of clinical *Klebsiella* isolates. The O1 polysaccharide backbone structure is known, but monoclonal antibodies raised against the O1 antigen showed varying reactivity against different isolates that could not be explained by the known structure. Reinvestigation of the structure by NMR spectroscopy revealed the presence of the reported polysaccharide backbone (glycoform O1a), as well as a previously unknown O1b glycoform composed of the O1a backbone modified with a terminal pyruvate group. The activity of the responsible pyruvyltransferase (WbbZ) was confirmed by western immunoblotting and in vitro chemoenzymatic synthesis of the O1b terminus. Bioinformatic data indicate that almost all O1 isolates possess genes required to produce both glycoforms. We describe the presence of O1ab-biosynthesis genes in other bacterial species and report a functional O1 locus on a bacteriophage genome. Homologs of *wbbZ* are widespread in genetic loci for the assembly of unrelated glycostructures in bacteria and yeast. In *K. pneumoniae*, simultaneous production of both O1 glycoforms is enabled by the lack of specificity of the ABC transporter that exports the nascent glycan, and the data reported here provide mechanistic understanding of the capacity for evolution of antigenic diversity within an important class of biomolecules produced by many bacteria.

## Significance

O antigens are cell-surface polysaccharides that provide validated targets for immunotherapeutic interventions to treat infections by increasingly antibiotic-resistant isolates of *Klebsiella pneumoniae*. This strategy requires detailed knowledge of O antigen structure, as well as the distribution of specific structures and potential antigenic epitopes in clinical isolates. Here, we show that the O antigen of the most prevalent *K. pneumoniae* serotype, O1, has two glycoforms. One form was established in the literature, but it can be produced together with a previously unrecognized form that possesses an additional terminal pyruvate residue. The findings are relevant for immunotherapeutic development but also offer important fundamental insight into bacterial polysaccharide assembly and possible mechanisms for antigenic diversification.

*Klebsiella pneumoniae* is a complex bacterial species comprising several phylogroups, including versatile nosocomial and community-acquired pathogens that cause infections in neonates, the elderly, and immunocompromised individuals (1). *Klebsiella* represents the “K” of the *ES****K****APE* group of important antimicrobial-resistant pathogens and is part of the carbapenem-resistant and extended-spectrum β-lactamase-producing Enterobacterales that appear on the World Health Organization’s critical threat list (reviewed in refs. 1 and 2). A recent study of global deaths in 2019 due to antimicrobial-resistant bacteria attributed 642,000 fatalities to *K. pneumoniae* (13% of the total and the third most prevalent species) (3). Its global distribution, high mortality rates, and health care burdens have led to a renewed interest in immunotherapeutic approaches to tackle *K. pneumoniae* (reviewed in ref. 4).

*Klebsiella* cell-surface polysaccharides [K-antigen capsular polysaccharides and lipopolysaccharide O antigen polysaccharides (OPSs)] are important for pathogen survival in the host, and they offer candidate targets for immunotherapeutic strategies. K antigens are potentially a complicated target because there are ~80 recognized structural types with even more predicted by sequence data (5–7). K-antigen diversity is more extensive in the so-called “classical” strains, which cause antibiotic-resistant infections in health care settings. However, two serotypes (K1 and K2) are particularly prevalent in the “hypervirulent” isolates responsible for community-acquired infections in immunocompetent individuals (reviewed in ref. 1), and glycoconjugate vaccines containing K1 and K2 polysaccharides are protective in murine infection models (8, 9). The smaller number of known O antigens has made them the focus of several investigations. The exact number of *Klebsiella* O serotypes is still being refined because the traditional serological classification of O antigens was hampered by lack of validated serotype-specific antisera, correlated with specific OPS structures. However, recent development of molecular (sequence-based) serotyping has provided insight into the extent of diversity of O serotypes in large isolate collections (10, 11). This has led to valuable online tools such as Kaptive Web (12) and Pathogenwatch (Kleborate) (13) for surveillance and serotype documentation. Available global data suggest that ~75 to 100% of clinical isolates belong to serotypes O1, “O2,” “O3,” and O5 depending on the isolate collection(7, 10, 11, 13–15). A recent study of isolates from bloodstream infections from a hospital in Chicago reported that 83% of 104 isolates belonged to O1, “O2,” and “O3” (15). Serotype O1 is typically the most abundant. It has been reported that antibodies against O antigens offer reduced protection against encapsulated *K. pneumoniae* isolates (particularly hypervirulent isolates) in murine models of infection, possibly due to capsule covering O antigen epitopes on the cell surface (9). Nevertheless, several studies have described protective anti-OPS antibodies in animal models of infection, with opsonophagocytosis involved (16–20). Furthermore, a multivalent O1, O2, O3, and O5 OPS-protein conjugate elicited antibodies that confer passive protection in infection models (21). In summary, active or passive immunization targeting *Klebsiella* polysaccharide antigens offers promising treatment options to tackle infections caused by increasingly antibiotic-resistant isolates.

**Fig. 1.**
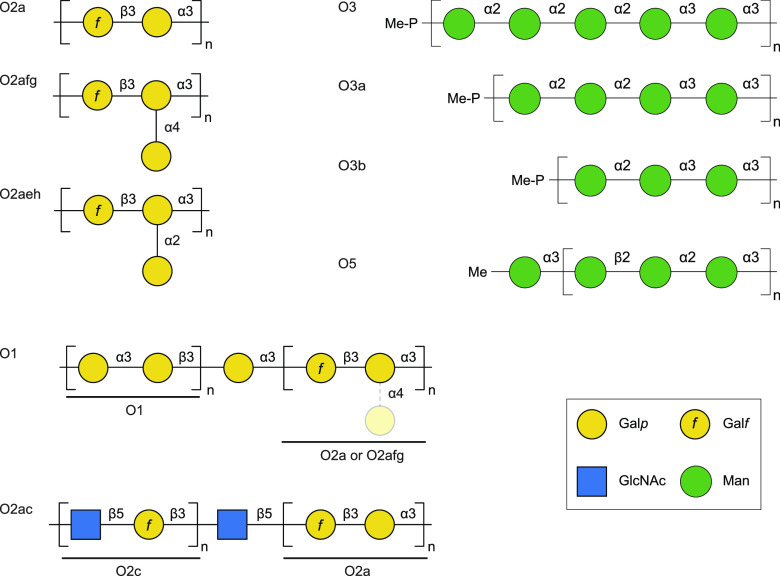
OPS repeat-unit structures reported from selected *K. pneumoniae* O-antigen serotypes. Only the sugar components are shown for O1 and “O2,” but nonstoichiometric O-acetylation can add further antigenic diversity. The polymannose OPSs possess methyl (O5) or methylphosphate (“O3”) moieties at their nonreducing termini. The OPS components are galactofuranose (Gal*f*), galactopyranose (Gal*p*), N-acetylglucosamine (GlcNAc), mannose (Man), methyl (Me), and phosphate (P). The chemical repeat unit structures are enclosed by parentheses.

The OPS structures of the clinically prevalent serotypes have been determined (Fig. 1). Serotype “O2” is actually a family of structures resulting from modification of the O2a main chain with sugar residues in different linkages, creating antigenically distinct subserotypes (O2aeh and O2afg) (22–25). Further diversification results from addition of another structurally distinct repeat-unit domain (O1 and O2c antigens) to the nonreducing reducing terminus of O2a (22, 26–28) or O2afg (29). Nonstoichiometric O-acetylation adds further minor epitopes in “O2” (23, 24). Serotypes O1 and “O2” are the most abundant and are frequently correlated with antibiotic-resistant isolates (11), although distribution of each serotype varies across patient age groups and *Klebsiella* phylogroup (13). Like “O2”, serotype “O3” is also a group of related structures (O3, O3a, and O3b), possessing both unique and cross-reactive epitopes (30); O3b seems to be the more prevalent subserotype in isolate collections (7, 30). The O3 and O5 antigens both possess nonreducing terminal nonglycose moieties (31–33), which are important in biosynthesis and surface presentation (see below). While sequence data are a powerful tool for serotyping, recognizing and interpreting sequence variations that may alter the activities of biosynthetic enzymes remains a challenge. For example, mutations in a multidomain glycosyltransferase underpin variation in the O3, O3a, and O3b serotypes (30, 34), but the outcome in polysaccharides structure is difficult to predict from individual mutations (30). Furthermore, a survey of “O2”-related genetic loci has revealed the influence of *IS*-element insertion sequences on the structure and production of OPS (35).

Immunotherapeutic approaches demand a clear understanding of OPS fine structure. In addition, contemporary vaccine production strategies involving expression of OPS in heterologous host backgrounds, or glycoengineering for in vivo glycosylation of carrier proteins, require correlation of precise structures with specific genetic sequences to generate production strains (36, 37). Here, we show that the clinically predominant O1 antigen exists as two different glycoforms. One is the established O1 glycan, while the other is a previously unrecognized OPS structure distinguished by possession of a nonreducing terminal pyruvate modification. In addition to the possible implications for immunotherapy, the findings reported here provide important insight into the potential for generating further antigenic diversity in *K. pneumoniae* and other bacteria.

## Results

### Identification of a Previously Unidentified O1 OPS Glycoform.

**Fig. 2.**
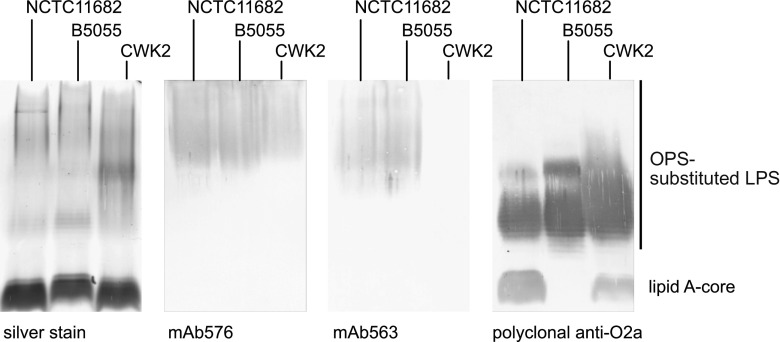
Western immunoblot analysis of LPS from serotype O1 representatives. The samples were prepared as whole-cell lysates. The SDS-PAGE was silver-stained, and the immunoblots were probed with the identified monoclonal and polyclonal antibodies. The difference in reactivity of the unabsorbed polyclonal Abs with the lipid A-core region in the LPS profile likely reflects known differences in outer core structures (38). The Abs were raised against a derivative of CWK2 which shares the type 1 core structure with NCTC1182 based on genome sequence (UGKQ01000007.1 and UGMM01000004.1), but B5055 has a type 2 core structure based on genome sequencing (CP072200.1) and lacks reactivity. Silver staining and western blotting were performed in triplicate with comparable results.

Reinvestigation of the O1 OPS structure was prompted by the analysis of the reactivities of two monoclonal antibodies (mAbs) originally raised against a protein–polysaccharide bioconjugate composed of protein AcrA glycosylated with O1 polysaccharide. Unexpectedly, the mAbs showed different patterns of western immunoblot reactivity against LPS in whole-cell lysates from well-studied *Klebsiella* strains NCTC11682 (O1:K2) and CWK2 (O1:K20^−^). mAb576 reacted with both lysates, while mAb563 only recognized NCTC11682 LPS, indicating that it was specific for a key epitope missing in CWK2 (Fig. 2). Identical O1 OPS chemical structures have been reported from two *K. pneumoniae* isolates: CWK2 (26) and B5055 (O1:K2) (27, 28), but a cell lysate from B5055 showed mAb reactivity comparable to NCTC11862. As expected from previous studies, polyclonal anti-O2a antibodies recognized OPS with shorter chain lengths present in all strains, while both mAbs reacted with high-molecular-weight species, which are known to contain the O1 repeat-unit structure (26).

**Fig. 3.**
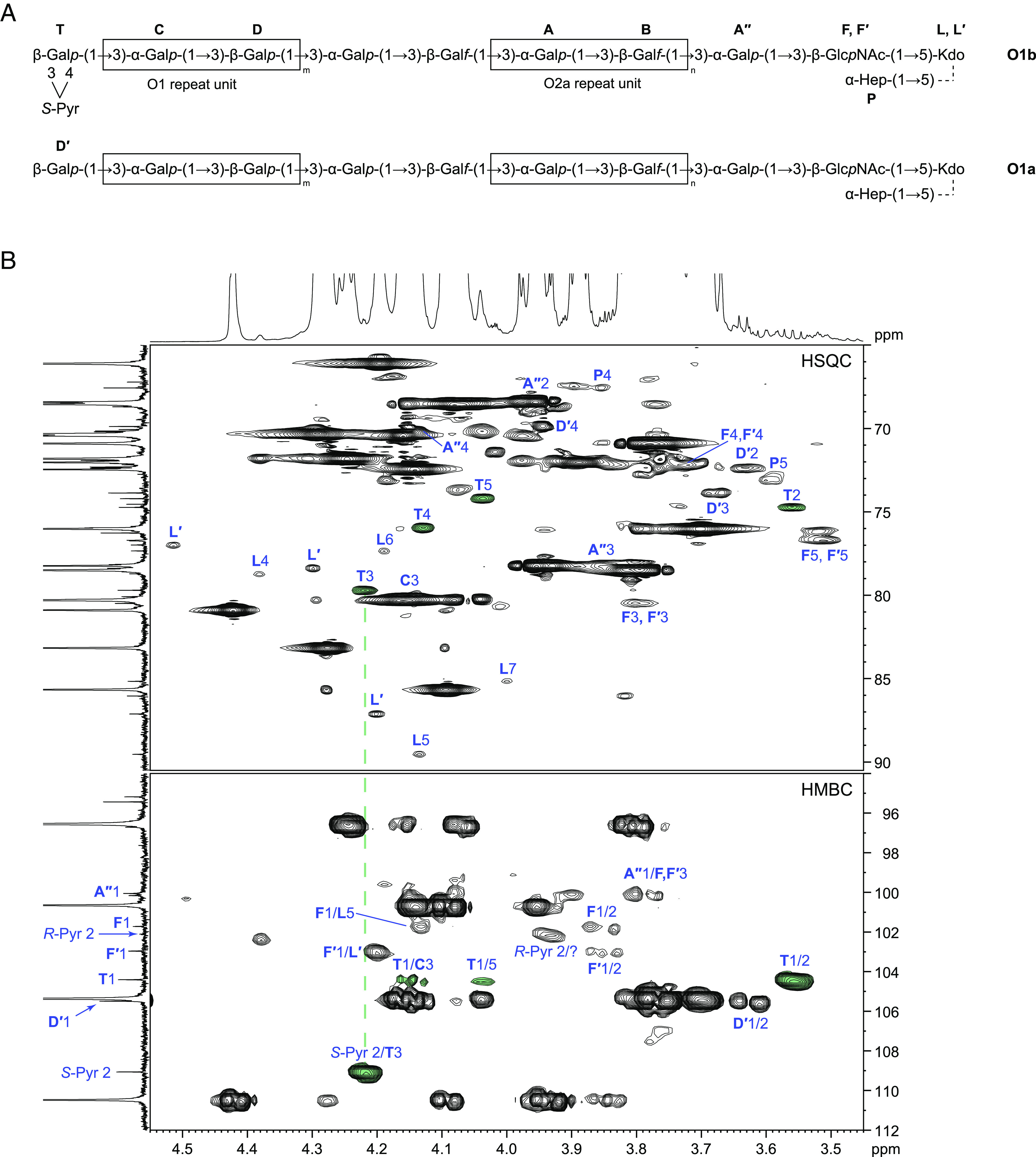
Structural determination of the two O1 OPS glycoforms. (*A*) Shows the final structures of the O1b (*Upper*) and O1a (*Lower*) glycans obtained from mild acid degradation of *K. pneumoniae* NCTC11682 LPS. The O2a and O1 glycan repeat units are boxed. The substitution of the outer core Kdo residue with Hep is nonstoichiometric. The sugar residue labeling follows assignments given in an earlier study of *K. pneumoniae* OPS terminal modifications (32). (*B*) Shows parts of ^1^H,^13^C HSQC (*Top*) and HMBC (*Bottom*) spectra of the OPS. The corresponding parts of ^1^H and ^13^C NMR spectra are shown along the horizontal and vertical axes, respectively. Correlations belonging to terminal β-Gal*p*3,4(*S*-Pyr) (residue **T**) are marked with green. Attachment of *S*-Pyr at position O-3 and O-4 of **T** was confirmed by HMBC correlation between ketal carbon of Pyr and **T** H-3 at δ 109.1/4.22. NMR was performed once.

To resolve the chemical basis for differential mAb563 immunoreactivity, the OPS from NTCC11682Δ*cps* was isolated by mild acid hydrolysis, and its structure was determined by 1- and 2-dimensional ^1^H and ^13^C NMR spectroscopy and compared to the reported O1 structure. Mild acid hydrolysis of *K. pneumoniae* LPS cleaves a labile linkage within the core oligosaccharide (32), and signals for sugar residues remaining at the reducing end of the mild acid-liberated OPS were consistent with the known type 1 core structure, where the OPS is ligated to a terminal 3-deoxy-d-*manno*-oct-2-ulosonic acid (Kdo) (Fig. 3*A*) (32, 39). As anticipated from the reported OPS structures, the major series of signals were assigned to the internal residues of the O2a disaccharide (residues **A**, **B**) and O1 disaccharide (residues **C**, **D**) repeat units that together comprise the established O1 OPS backbone (*SI Appendix*, Table S1). The minor signals originating from the residues at the reducing and nonreducing ends of the glycan were clearly visible in the HSQC-TOCSY spectrum (*SI Appendix*, Fig. S1), and most were assigned (*SI Appendix*, Table S1). In addition, the NMR data revealed the presence of two pyruvic acid ketal residues (Pyr) in an ~ 1:5 ratio, which were not reported previously.

The major Pyr methyl group at δ_H_ 1.61 showed HMBC correlations with the carboxyl carbon C-1 (δ 178.6) and ketal carbon C-2 (δ 109.1); the latter is typical for 1,3-dioxalane (five-membered) rings (40). The HMBC spectrum also showed a correlation at δ 109.1/4.22, identifying the location of the Pyr residue (Fig. 3*B*). The signal at δ 4.22 was assigned to H-3 of a nonreducing terminal β-Gal*p* (residue **T**). Correlations from **T** C-1 to H-1−H-4 were visible in the HSQC-TOCSY spectrum (*SI Appendix*, Fig. S1), and the protons within the **T** spin system were assigned by tracing well-isolated H-1/H-2 and H-2/H-3 COSY connectivities. The **T** H-5/C-5 signal was found from **T** C-1/H-5 HMBC correlation and confirmed by **T** C-5/H-4 and **T** C-5/H-6a, b correlations in the HSQC-TOCSY spectrum. The **T** C-1/**C** H-3 correlation in the HMBC spectrum confirmed the **T**-(1→3)-**C** linkage (Fig. 3*B*). The downfield displacement of the signals **T** C-3 and C-4 [relative to their positions in unsubstituted β-Gal*p* (41), combined with the Pyr C-2/**T** H-3 HMBC correlation, demonstrated that the predominant Pyr is ketal-linked to **T** O-3 and O-4. The chemical shifts of the pyruvic methyl group (δ 1.61/24.6) are consistent with an *S* configuration for the ketal carbon (40). This was confirmed by a 1D-ROESY experiment with selective excitation of the Pyr methyl group signal, which showed the expected sole correlation with **T** H-2 (*SI Appendix*, Fig. S2*A*) (42). The ketal carbon of the minor Pyr resonated at δ 102.1, which is typical for a 4,6-pyruvate (six-membered) dioxane ring (40), and the chemical shifts of the methyl group (δ 1.48/26.4) demonstrated an equatorial position (corresponding to *R* configuration if Pyr is attached to a Gal*p* residue) (40). An attempt to unequivocally establish the location of the minor Pyr ketal was made, based on the observed HMBC correlation at δ 102.1/3.94, but the corresponding sugar spin system could not be assigned due to low signal intensities and overlap issues.

The NMR spectra also contained the signals for an unsubstituted nonreducing terminal β-Gal*p* residue (**D′**). The **D′→C** linkage was independently confirmed by a **D′** H-1/**C** C-3 HMBC correlation and a 1D ROESY experiment with excitation of the anomeric proton **D′** H-1, that showed correlations with **C** H-2, H-3, and H-4. The ratio between pyruvylated and nonpyruvylated β-Gal*p* residues is ~ 1:1 based on integrating HSQC peaks **T**2, **T**4, and **D′**2, **D′**3 (Fig. 3*B*). This is only an estimate because the standard HSQC experiment is not quantitative and overlapping signals in the ^1^H NMR spectrum precluded a more accurate assessment. The collective data identify a structural element for serotype O1; a 3,4-linked *S*-pyruvic acid ketal, which caps some of the nonreducing terminal β-Gal*p* residues of the O1 repeat unit. The presence of a nonpyruvylated β-Gal*p* may reflect the natural expression of modified and unmodified OPS chains (see below). However, the initial degree of pyruvylation may be higher because the ketal group is acid-labile and may be partially removed during mild acid hydrolysis of LPS to release the OPS.

### Synthesis of the O1b Terminus Requires the WbbZ Pyruvyltransferase.

Biosynthesis of the O1 antigen backbone requires two genetic loci (*SI Appendix*, Fig. S3). The main locus (*rfb*^2a^) encodes the enzymes necessary for biosynthesis of the O2a portion of the chain (43), while a second unlinked O1 locus is required only for addition of the O1 polysaccharide onto an O2a acceptor (44). The O1 locus contains two divergently transcribed genes, *wbbY* and *wbbZ* (45). WbbY is the O1 polymerase and contains two glycosyltransferase (GT) catalytic modules, generating the two linkage types in the O1 repeating-unit structure (44). The sequence of WbbZ predicts a pyruvyltransferase by BLAST (45) (see below), but its participation in OPS production has not been investigated because the presence of a terminal pyruvate residue was unknown until now. Furthermore, *wbbZ* is disrupted by an *IS*5 sequence in the prototype *K. pneumoniae* CWK2 strain used in most biochemical studies (44). Notably, the constructs used for generating the glycoengineered bioconjugate antigen for mAb production encoded an intact WbbZ, implicating this protein as the source of the terminal pyruvate residue.

**Fig. 4.**
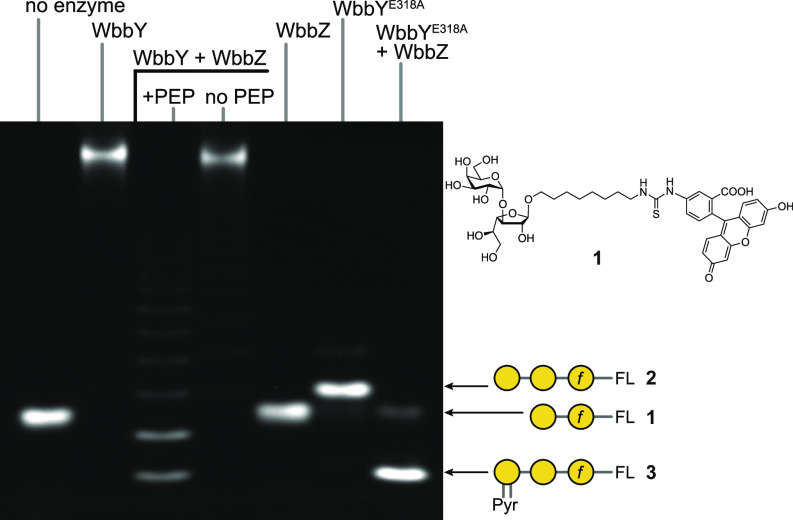
Validation of the pyruvyltransferase activity of WbbZ. WbbY^1-722^ catalyzes the polymerization of the O1 antigen using compound **1** (shown to the right) as an acceptor, and inclusion of WbbZ results in premature polymerization termination that is dependent on the presence of PEP. WbbY^E318A^ transfers a single β-Gal*p* residue onto **1** resulting in trisaccharide compound **2.** Inclusion of WbbZ with WbbY^E318A^ results in a fast-migrating species, consistent with the added charge of pyruvate. The identities of the various compounds were determined by mass spectrometry (*SI Appendix*, Fig. S4) and NMR spectroscopy (*SI Appendix*, Fig. S5). Reactions were performed three times with similar results. FL is the fluorescent aglycone.

An in vitro strategy was taken to unequivocally demonstrate that WbbZ possesses pyruvyltransferase activity and investigate the enzyme’s specificity. As reported previously, His_6_-WbbY^1-722^ extends the synthetic acceptor compound **1** (representing the O2a disaccharide with a terminal Gal*p*) by adding a high-molecular-weight O1 polymer in the presence of its donor substrate, UDP-Gal*p* (Fig. 4) (44). Addition of His_6_-WbbZ (from NCTC11682) to a WbbY reaction mixture resulted in phosphoenolpyruvate (PEP)-dependent reduction of the size of the reaction products, suggesting premature termination of O1 polymerization in vitro (Fig. 4).

WbbZ was unable to modify the terminal α-(1→3)-Gal*p* residue in compound **1** in the absence of WbbY (Fig. 4), implicating the β-Gal*p* residue in the O1 repeat-unit as the site of modification, consistent with the structure of the natural product. To verify both the addition of pyruvate and its exact linkage, a trisaccharide acceptor (compound **2**) was first obtained by chemoenzymatic synthesis in a reaction containing compound **1** and WbbY^E318A^, which possesses a catalytic-site mutation inactivating the α1,3-Gal*p* GT module (44). WbbZ converted compound **2** to a faster migrating product **3.** The structures of compounds **2** and **3** were determined by ESI mass spectrometry (*SI Appendix*, Fig. S4) and by NMR spectroscopy (*SI Appendix*, Table S1). Compound **2** consisted of the expected trisaccharide linked to the aglycone. HSQC and HMBC spectra of **3** contained signals for an *S*-Pyr residue at δ_H_/δ_C_ 1.59/24.7 (methyl group), δ_C_ 109.0 (ketal carbon C-2), and 178.7 (carboxyl carbon C-1). Comparison of the HSQC spectra of **2** and **3** demonstrates that that the signals for terminal β-Gal*p* (residue **T**) were mostly affected by the Pyr modification (*SI Appendix*, Fig. S5 *A* and *B*). In particular, the signals for **T** C-3 and C-4 were shifted downfield to δ 79.7 and 76.0, compared to their positions at δ 73.7 and 69.7 in **2**, in agreement with the attachment of Pyr to positions **T** O-3 and O-4. The location of Pyr was independently confirmed by correlation between **T** H-3 and Pyr C-2 at δ 4.13/109.0 in the HMBC spectrum. Finally, the *S* configuration of Pyr was confirmed by a 1D ROESY experiment (Fig. 2*B*). Notably, no other minor Pyr signals were present in the HSQC or HMBC spectra of purified compound **3** (^1^H NMR spectroscopy was not useful in this case because the signal of an *R*-Pyr methyl group (if present) would overlap with the broad signal for methylene protons of the FITC linker (*SI Appendix*, Fig. S2 *A* and *B*). Although we cannot exclude the possibility that trace amounts of side product(s) were lost during size-exclusion chromatography purification of **3**, that amount would not be sufficient for 2D NMR spectroscopy. Thus, the only WbbZ pyruvyltransferase activity detected and structurally characterized in the in vitro products is the 3,4-linked *S*-Pyr. The origin of minor signals for the 4,6-linked *R*-Pyr in the NMR spectra of native OPS remains unknown. We hypothesize that it might be an artifact resulting from migration of Pyr residues during isolation of the OPS from bacterial cells and may not be present in the LPS in vivo.

The connection between WbbZ-mediated pyruvylation and the epitope recognized by mAb563 was established by the conversion of *K. pneumoniae* CWK2 to mAb563 reactivity following transformation with the plasmid (pWQ1113) carrying a wild-type copy of *wbbZ* (*SI Appendix*, Fig. S6). Notably, when NCTC11862 was transformed with the same plasmid, the abundance of mAb563-reactive OPS increased, suggesting that the WbbZ-mediated modification is incomplete in the wild-type strain. This is consistent with the identification of both modified and unmodified OPS structures in purified LPS.

### Distribution of *wbbZ* in *K. pneumoniae*.

The reported OPS structure of *K. pneumoniae* B5055 contains only the O1a antigen (27, 28) but the mAb563 reactivity is contradictory. Examination of the B5055 genome sequence (GenBank CP072200.1) revealed an intact *wbbZ* gene essentially identical to NCTC11682. The published ^13^C NMR spectra for B5055 OPS showed no evidence of resonances corresponding to Pyr C-2, although a combination of low degree of pyruvylation, long OPS chain length, and insufficient scans could result in a signal-to-noise ratio too low to detect minor modifications. Unfortunately, the part of the ^1^H NMR spectrum that would contain the resonances for the Pyr methyl group was not presented. Thus, the absence of Pyr in the original B5055 OPS structure could result from it being overlooked as a minor signal in the spectra or loss of the Pyr modification during preparation of B5055 OPS for structural studies. The prior investigation did not benefit from the genetic and mAb data described here to point the authors to further complexity.

The difference in genetic data for *K. pneumoniae* CWK2 and B5055 prompted a broader analysis of *wbbZ* status, by exploiting a blastn search of the collection of 1,717 geographically diverse *Klebsiella* isolates from the European Survey of Carbapenemase-Producing Enterobacteriaceae (46) (Dataset S1). Of these, 618 isolates contained both *wbbY* and *wbbZ*, and all but one of this group also possessed the O2a biosynthesis genes, indicating that almost all have the potential to produce both the 1a and 1b antigens. The exception (19646_2#3) contains a *K. pneumoniae* serotype O4 cluster instead of *rfb*^O2a^ and so would be unable to produce the O1 antigen. Of the 624 isolates possessing *wbbZ*, only six lacked a corresponding intact *wbbY* gene. Conversely, only three of 621 isolates with *wbbY* lacked an intact *wbbZ* and this was due exclusively to premature stop codons; this group of isolates appears confined to producing only the O1a antigen. A total of 29 isolates showed partial matches to *wbbZ* (ranging from 18 to 79% of the gene sequence length) and were classed as *wbbZ*-negative; three of these lacked *wbbY*. Given the arbitrary selection of *K. pneumoniae* O1:K20 as a model for genetic and structural analysis, it was surprising to see so few isolates with similarly disrupted *wbbZ* genes and none with the same insertion sequence observed in CWK2. That insertion did not arise during selection of the *cps* mutation in CWK2 because the parent (889/50 = NCTC9140, which is a reference strain for the K20 antigen) carries the same insertion (GenBank accession UGKQ01000007.1). Collectively, these data suggest that there is a strong correlation between possession of *wbbZ*, *wbbY*, and the O2a gene cluster in these clinical isolates, and almost all “O1” isolates may possess both the O1a and O1b glycoforms. This observation should be considered in immunotherapeutic development.

A gene (*wbmX*) encoding a homolog of WbbZ was also found in *K. pneumoniae* serotype O2c. Like *K. pneumoniae* O1, the O2ac antigen is also attached to the nonreducing end of an O2a chain (Fig. 1). The *wbmX* gene is located in a three-gene locus, together with *wbmV* and *wbmW* (*SI Appendix*, Fig. S7*A*). In vitro reconstitution of O2c biosynthesis has illustrated that WbmV and WbmW are GTs needed to produce the O2c glycan backbone (44). WbmX was originally predicted to be a GT-B-fold glycosyltransferase enzyme, but it shares the conserved motifs found in the functionally characterized pyruvyltransferases in a multiple sequence alignment (*SI Appendix*, Fig. S8; see below). This prompted a reinvestigation of the NMR data for the O2c structure from the LPS of *E. coli* containing *rfb*^2a^ and the *wbmVWX* locus (47), with the hypothesis that it too might include an overlooked terminal pyruvate. Indeed, an expanded HSQC spectrum contained a characteristic methyl peak at δ_H_/δ_C_ 1.55/23.1, that showed HMBC correlations with the ketal carbon (δ_C_ 109.0) and carboxyl carbon (δ_C_ 178.8) of pyruvate (*SI Appendix*, Fig. S7*B*). The exact linkage of the pyruvate could not be identified from the available NMR data, but it is likely that it involves a five-membered ring structure from chemical shift data. The precise structure was not pursued further because it was not central to the goals of this study. Nevertheless, the sequence and OPS structure data all indicate that WbmX is a previously unrecognized pyruvyltransferase.

### Distribution of *wbbY-wbbZ* Genes beyond *K. pneumoniae*.

In *K. pneumoniae* O1, the *wbbY* and *wbbZ* genes are located between transposable elements (45), suggesting acquisition by lateral gene transfer. A tblastn search indicated that *wbbYZ* genes are found in other bacteria ranging from virtually identical examples in *E. coli*, *Raoultella*, and *Citrobacter* to more distantly related in representatives from *Pseudochrobactrum, Lysobacter,* and *Acetobacter* (*SI Appendix*, Table S2). Notably, in the *E. coli* isolates, the *wbbYZ* genes are frequently located nearby a complete *rfb*^2a^ gene cluster, although the chromosomal locations for the loci vary among isolates. In one case, these genes are found on a plasmid together with the *gmlABD* genes, which direct production of the O2aeh antigen in *K. pneumoniae* (47). However, in some isolates, the *rfb*^2a^ cluster possesses a deletion or is entirely absent. In most isolates, the genome sequences provided evidence of a serotype-specific *rfb* operon at the typical location (near *his* and *gnd*) in *E. coli*, which directs the synthesis of known *E. coli* O antigens. Therefore, in principle, many of these isolates may coexpress two different O antigens, one cross-reacting with *K. pneumoniae* O1. This may explain the previously reported reactivity of polyclonal *E. coli* O19 serotyping antibodies with *K. pneumoniae* O1 LPS (48).

**Fig. 5.**
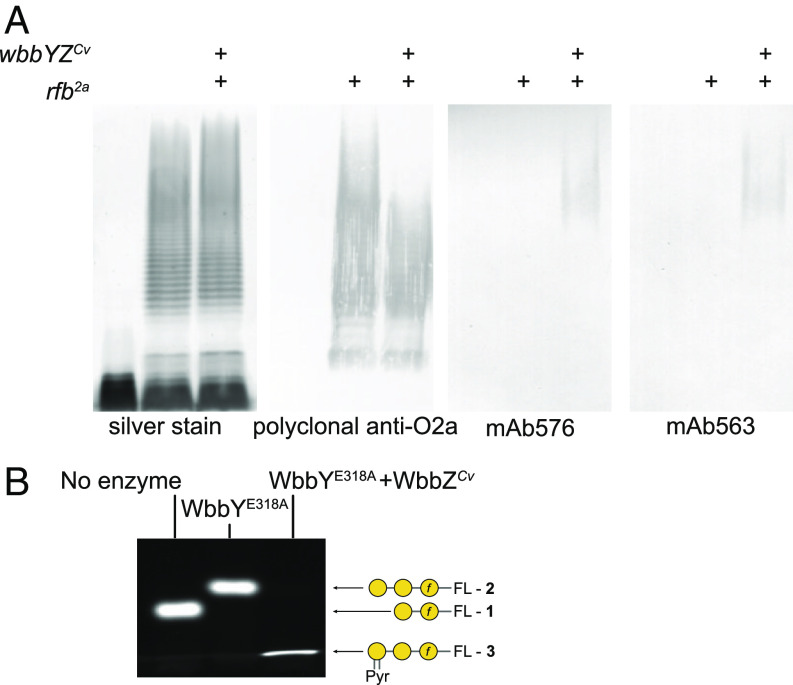
Activity of WbbY and WbbZ proteins encoded by a bacteriophage belonging to the *Caudovirales*. (*A*) Western immunoblots demonstrating synthesis of the O1a and O1b antigens in *E. coli* DH5α. The *Caudovirales wbbYZ^Cv^* sequences were identified from accession BK049963.1 in the BLAST search. The genes were synthesized, cloned in plasmid pWQ1115, and transformed into *E. coli* DH5α, together with plasmid pWQ288 carrying the *rfb*^2a^ locus (52). LPS in whole-cell lysates was probed using the identified antibodies. Silver staining and western blotting were performed twice with the same results. (*B*) Cell-free lysates containing *K. pneumoniae* WbbY^E318A^ and WbbY*^Cv^* were combined and reacted with compound 1 in the presence of UDP-Gal*p* and PEP. The identities of the reaction product (and control reaction products) shown were confirmed by MS (*SI Appendix,*Fig. S9). The same PAGE profile was obtained in three independent experiments, products shown were confirmed by MS. The tight band observed with the PAGE migration of **3** is a result of comigration with the loading dye.

Bacteriophages play an important role in the diversification of bacteria surface polysaccharides, acting as agents of selective pressure as well as a source of modification genes. Notably, *wbbY* and *wbbZ* genes were also found in the genome of a phage belonging to the *Caudovirales* (GenBank accession BK049963.1) resulting from a study of the metagenomes from individuals with chronic diseases (49). Phages are the most abundant biological entity on earth (50) and tailed dsDNA phages in the *Caudovirales* constitute the largest representative group. Representatives that exploit cell surface polysaccharides as receptors are well documented in *K. pneumoniae* and other bacteria (51). To test whether the *Caudovirales* genes encode functional enzymes, the locus was synthesized and inserted in plasmid pWQ1115. This plasmid was then used to transform *E. coli* DH5α containing pWQ288 (which carries the *rfb*^2a^ locus conferring O2a OPS production) (52). Cotransformation with pWQ288 and pWQ1115 conferred reactivity against both mAb576 and mAb563 indicating production of the O1a and O1b antigens (Fig. 5*A*). Furthermore, in vitro reactions with cell-free lysate containing the *Caudovirales* WbbZ*^Cv^* protein generated compound **3** from compound **2** (Fig. 5*B*), corroborating the activity and acceptor specificity of the pyruvyltransferase. Several other glycan modifying processes have been described in bacteriophages, and they are thought to alter the structures of phage receptors, influencing the outcome of subsequent phage–host interactions (reviewed in ref. 53).

### Phylogeny of Bacterial Pyruvyltransferases for Polysaccharide Modification.

**Fig. 6.**
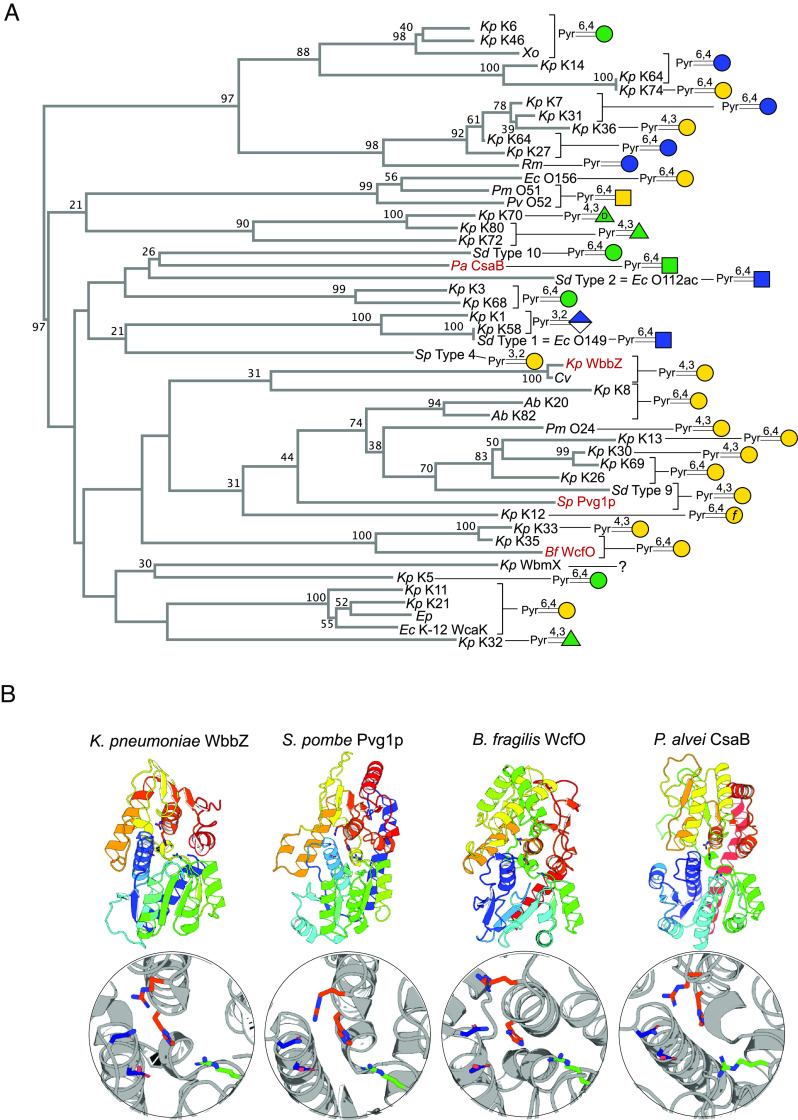
Phylogenetic analysis of known and predicted glycan pyruvyltransferases and structures of Pvg1P and WbbZ. The phylogenetic tree was established by a multiple sequence alignment (*SI Appendix*, Fig. S8) of WbbZ orthologs encoded in polysaccharide-biosynthesis loci from bacteria with known polysaccharide structures (*SI Appendix*, Table S3) MAFFT was used to generate the alignment and build the tree, and bootstrap values greater than 20 (from 100 iterations) are shown. The pyruvate linkage is shown (omitting absolute configuration). Species names are abbreviated as follows: *Ab; Acinetobacter baumannii, Bf; Bacteroides fragilis, Cv; Caudovirales sp., Ep; Erwinia pyrifoliae, Ec; Escherichia coli, Kp; Klebsiella pneumoniae, Pa; Paenibacillus alvei, Pm; Proteus mirabilis, Pv; Proteus vulgaris, Rm; Rhizobium meliloti, Sp; Schizosaccharomyces pombe, Sd; Shigella dysenteriae, Sp; Streptococcus pneumoniae, Xo; Xanthomonas oryzae.* The entries in red in (*A*) have a solved structure or were modeled using AlphaFold and are shown in (*B*). Crystal structures for WbbZ (PDB: 6x1l) and Pvg1p (PDB: 5ax7) are shown, and WcfO and CsaB are AlphaFold models. Structures are colored in rainbow from blue (N terminus) to red (C terminus) to facilitate comparison. The predicted active site is expanded, and the residues shown as sticks are some of those conserved in the multiple sequence alignment in *SI Appendix*, Fig. S6.

Pyruvate substitutions occur frequently in different types of bacterial glycoconjugates and in some eukaryotes (reviewed in ref. 54). After the characterization of WbbZ, we sought to survey the landscape of orthologous pyruvyltransferases involved in glycan biosynthesis. Due to the lack of global sequence similarity observed among pyruvyltransferases, the Carbohydrate Structure Database (CSDB; ref. 55) was used to identify glycans with known pyruvate modifications produced by bacteria with a deposited genome sequence. The corresponding glycan clusters were then manually searched to identify 49 genes encoding candidate pyruvyltransferase enzymes (*SI Appendix*, Table S3). The limited number of entries obtained exemplifies the general disconnect between structural and genetic information and the available information being heavily weighted toward pathogens. The majority of the candidates (25 in total) are implicated in modifying *K. pneumoniae* capsular polysaccharides. Phylogenetic analyses identified three main clades of enzymes each containing a biochemically validated enzyme (Fig. 6*A*). CsaB modifies N-acetylmannosamine residues in the secondary cell wall polysaccharides of *Paenibacillus alvei* (56) and WcfO, which modifies Gal*p* residues in CPSA capsular polysaccharide from *Bacteroides fragilis* (57). WbbZ is positioned in a third clade, along with Pvg1P from *Schizosaccharomyces pombe*, which pyruvylates terminal β-(1→3)-Gal*p* residues in N-linked glycans (58). This clade includes other enzymes that modify capsular polysaccharides (e.g. from *K. pneumoniae* and *Acinetobacter baumannii*) and OPS (e.g. from *Shigella dysenteriae* and *Proteus mirabilis*). Interestingly, the WbmX enzyme from *K. pneumoniae* O2ac does not map to the same clade as WbbZ from *K. pneumoniae* O1. Ketal-linked pyruvate modifications of bacterial polysaccharides are typically placed across the 2, 3-, 3, 4- and 4, 6- positions of sugars, and among those with recorded structures, modifications of galactose are most abundant (Fig. 6*A*). However, the clades do not segregate along the lines of either the linkage position or the modified sugar. Despite many of the proteins being from *K. pneumoniae*, and the proteins catalyzed similar or identical pyruvylation reactions, their global identities were low as evidenced by a multiple sequence alignment (*SI Appendix*, Fig. S8). For example, the putative *K. pneumoniae* K35 and K74 orthologs both perform 4,6-*R*-pyruvate modifications of Gal*p,* but they share only 14% identity with 69% coverage.

The relatively low levels of global similarity shared with other enzymes limited informative conclusions from the full-protein alignments, but the multiple sequence alignment did identify some conserved motifs (*SI Appendix*, Fig. S8). These are N^29^xG^31^D^32^, R^121^xxx(S/T), and H^226^ (numbers correspond to WbbZ). Crystal structures of Pvg1p (PDP: 5AX7; ref. 59) and WbbZ from *K. pneumoniae* (PDB: 6X1L) have been solved, revealing substantial structural similarity (Fig. 6*B*). In WbbZ, 10 α-helices and 10 β-strands contribute to two α/β/α domains resembling Rossmann domains in a glycosyltransferase GT-B fold. DALI searches for relevant proteins structurally related to WbbZ revealed Pvg1p (5AX7) as the top hit, with 19% identity and a Z-score of 22.1 using 243 of the 329 residues. The remaining hits contained low sequence identity and were dominated by structures of UDP-GlcNAc 2-epimerases, presumably due to the shared Rossmann fold, and were therefore not physiologically relevant. Pvg1p was previously modeled with PEP and β-Gal-pNP, predicting that both would bind in a positively charged cleft between the two Rossmann domains. In the model, R217 and R337 directly interacted with the carboxylate and phosphate of PEP respectively, while D106 made hydrogen bond contact with Gal and a D106A mutant of Pvg1P showed almost no activity (59). The positioning of these residues is conserved in the WbbZ structure (Fig. 6*B*), consistent with a similar mode of PEP binding in WbbZ and Pvg1P. Furthermore, AlphaFold modeling shows similar positioning of these conserved residues in WcfO and CsaB (Fig. 6*B*), suggesting that the general structure of this proposed PEP-binding site is conserved among the orthologs examined.

## Discussion

Serotype O1 is abundant in large collections of *Klebsiella* clinical isolates and can contribute as many as 50% of the isolates, depending on the study (7, 11, 13). As a result, O1 is an essential consideration for any immmunotherapeutic strategy targeting antibiotic-resistant *K. pneumoniae*. The discovery of a previously unrecognized O1 glycoform (O1b) provides an additional epitope for consideration in these approaches, and its importance is underscored by the discovery that most O1 isolates in a diverse collection possess the genes necessary for its synthesis. In this study, an antigen synthesized in WbbZ-proficient cells was used to generate two rodent MAbs that recognize OPS chains differing in the presence (or not) of terminal pyruvate residues. However, it is unknown whether the presence of pyruvate in the antigen skews the immune response to particular epitopes or alters the outcome with respect to functional (neutralizing) antibodies. These are important questions now requiring further investigation.

Although the existence of nonreducing terminal modifications of *Klebsiella* O3 and O5 (Fig. 1) was first reported 50 y ago (31, 60), details of the terminal structures, the broader distribution of such modifications, and their potential physiological importance are relatively recent discoveries. Such modifications may have been overlooked in some earlier studies that focused primarily on structures of glycan repeating units, Biosynthesis of the O3 and O5 OPSs has not been studied directly in *K. pneumoniae*, but the process has been described in detail in *E. coli* O9 and O8, which produce identical OPS structures, resulting from horizontal transfer of the corresponding genetic loci between these species (61). All known *Klebsiella* OPSs are assembled in the cytoplasm and exported by an ATP-binding cassette (ABC) transporter to the periplasm, prior to ligation to lipid A-core and translocation of the completed LPS to the outer membrane (53). Nonreducing terminal methyl (O5) and methylphosphate (O3) residues (Fig. 1) terminate polymerization of the OPSs. Based on the *E. coli* O9a prototype, a coiled-coil structure separates the catalytic site(s) of the termination enzyme(s) from the membrane-proximal OPS polymerase, so termination only occurs once the nascent OPS chains reach a length sufficient to span the distance (62–65). The distribution of OPS chain lengths established by this process is preserved in the final LPS because the ABC transporter contains a carbohydrate-binding module (CBM) that recognizes the terminal structure as a requirement for export; there is no export of shorted uncapped chains (66–68). The structural basis of this mode of transport has been described (69–71). Furthermore, a bioinformatics approach indicated that similar assembly and termination systems exist in other *Klebsiella* serotypes (72, 73), as well as in other bacterial genera possessing different types of surface glycans (74). Methyl and methylphosphate terminal residues dominate in the candidate systems, but two *Klebsiella* serotypes (O4 and O12) possess terminal Kdo residues (32). Other bacteria (e.g., *Burkholderia vietnamiensis* G4 and *Pseudomonas protegens*) offer candidates for chain-termination and chain-length regulation using pyruvate, but the critical validating biochemical evidence is not yet available.

The tight integration of chain termination and transport for these glycans creates fidelity in the surface-presented product, and chain length distribution is important in protection against host defenses (53). However, it also imposes a limit on antigenic diversification; any changes in synthesis enzymes must preserve terminal chemistry in the absence of accommodating changes in the exporter. Based on data from the *E. coli* O9 equivalents, “O3” variant structures result from mutations within the polymerase (WbdA) that affects the number of residues transferred by the α1,2-Man-specific GT module (34, 75) (Fig. 1); these changes do not influence termination and can be accommodated by the same exporter. Diversification by addition of GT modules for backbone synthesis or addition of side-chain residues is not evident in these and related serotypes because it would potentially compromise termination and export.

In contrast, biosynthesis of the O1 and O2 antigens does not use the same chain-length regulation-export coupling process, and the relaxed substrate specificity of the conserved ABC transporter in these serotypes offers more possibilities. In recombinant bacteria, the O2a transporter can even export a terminated O3a glycan (52). This flexibility is essential for facilitating the observed diversification of the O1 and O2 antigen groups (Fig. 1) by the addition of side-chain sugars and acetate groups, elongation by the addition of a polysaccharide with a different repeat-unit structure, and (now) a terminal pyruvate, without any apparent cost to the OPS transport. Therefore, a different approach is needed to establish the chain-length distribution profile in LPS molecules. The ability of the O1 polymerase to produce long OPS chains is evident in the in vitro experiments above, and the same is true of the O2a polymerase, WbbM (43). In the cell, the native O2a OPS chain length distribution requires the obligatory coupling of chain extension and export, indicating that the relative activities of these processes are critical (52). This results in a wide range of OPS chain lengths seen in SDS-PAGE and typical in OPS systems that lack a terminal moiety-CBM mechanism. Although the pyruvate modification (reflected in mAb563 reactivity) is confined to the longer O1 OPS chains, overexpression of WbbZ in vivo has only a modest effect on the pattern, and its absence in *Klebsiella* CWK2 does not lead to a substantial increase in chain lengths. The pyruvate addition in serotype O1 (and O2c) can therefore be viewed as contributing an epitope to antigenic diversity without affecting other facets of the assembly process. The only other comparable example is provided by *Vibrio cholerae* O1, where a terminal 2-O-methyl group present in serogroup Ogawa distinguishes it from Inaba (76). Although the underlying biochemistry has not been investigated, mutations in the methyltransferase eliminate the terminal modification without preventing export of Inaba OPS (77). The ABC transporter lacks a CBM module, but the extent of its substrate specificity is unknown. While both forms are immunogenic and generate protective antibodies, it has been suggested that the preferred live oral vaccine would be the Hiko variant that offers a combination of capped and uncapped chains (78).

The observation that isolates lacking *wbbZ* do exist, and the understanding that modification does involve a draw on PEP, a central and heavily regulated metabolic intermediate (79), raises the questions of why the modification is retained and whether it offers a selective advantage in the clinically prevalent *K. pneumoniae* O1. Pyruvylation does not influence the structure of the main chain, unlike side-chain addition which has a profound effect on O2a backbone conformation (80) resulting in a substantial change in reactivity against antibodies recognizing the unmodified backbone (47). Humans produce large quantities (~1% of the total antibody content in some estimates) of “anti-Gal” antibodies against the α-Gal*p*-(1→3)-Gal*p* epitope, which is not present in the human glycome. These antibodies bind to bacterial surface molecules, including the LPS of some *Klebsiella* isolates (with unidentified serotypes), leading to speculation that galactose-containing OPS may be recognized (81). Furthermore, the binding of these antibodies can influence the activation of the alternative complement by some bacteria (82). However, the OPS structure data presented here show that the *Klebsiella* O1 OPS glycoforms have terminal pyruvate or β-Gal residues, suggesting that both should avoid recognition by abundant anti-Gal antibodies. The NMR data for in vitro products synthesized by the WbbY polymerase also terminate in β-Gal residues (44). Consistent with the glycan structures, no reactivity was observed in western immunoblots of *K. pneumoniae* O1 LPS probed with validated anti-Gal MAbs (*SI Appendix*, Fig. S10). Human galectin-3 recognizes β-Gal epitopes in the LPS of bacteria and results in killing of *K. pneumoniae* CWK2 (83, 84). How pyruvylation influences OPS recognition by this (and potentially other) galectins represents another question for further investigation. Finally, Pvg1P-mediated pyruvylation of α-Gal residues introduces a negative charge and creates a terminal moiety that is not expressed in humans and possesses sialylation-like lectin-binding properties (59). While the collective data indicate that *K. pneumoniae* O1 produces a mixture of capped and uncapped chains when grown in culture, it remains to be established whether the extent of capping varies in the host and whether the modification aids that pathogen by influencing interactions with host factors.

## Materials and Methods

Detailed methods are provided in *SI Appendix*.

### Bacterial Strains and Growth Conditions.

Bacterial strain genotypes and source details are provided in *SI Appendix*, Methods. All strains were grown at 37 °C in Luria Bertani medium unless otherwise specified.

### Antibodies and Western Immunoblotting of LPS.

Monoclonal antibodies were generated by GenScript against an O1 polysaccharide: protein conjugate antigen produced by in vivo N-glycosylation. O1 antigen synthesis in the glycoengineered strain was achieved using the O2a biosynthesis locus (*SI Appendix*, Fig. S3) from *K. pneumoniae* NUHL24835 (GenBank CP014004) and the *wbbY-wbbZ* O1 locus from *K. pneumoniae* AKPRH07048 (GenBank LT174607). Rabbit anti-O2a antibodies were described previously (48), and a validated mAb recognizing the α-Gal-1,3-Gal epitope was obtained from Enzo Life Sciences (https://www.enzolifesciences.com/ALX-801-090/alpha-gal-epitope-galalpha1-3galbeta1-4glcnac-r-monoclonal-antibody-m86/). Antibody reactivities were assessed by western immunoblotting, exploiting SDS/proteinase K-treated whole-cell lysates.

### Isolation of OPS and Determination of Its Structure.

LPS was extracted from cells using a standard hot aqueous phenol method (38), and OPS was released from purified LPS by mild acid hydrolysis and isolated by size exclusion chromatography. For NMR spectroscopy, OPS was deuterium-exchanged, and ^1^H and ^13^C NMR spectra were recorded at 50 °C (OPS) in D_2_O. Two-dimensional NMR spectra were obtained using standard Bruker software.

### Determination of WbbZ Enzyme Activity.

His_6_-tagged enzyme derivatives were purified from lysates of *E. coli* TOP10 transformants by Ni^2+^-NTA agarose chromatography. Standard analytical in vitro reactions were performed in 100 mM Na HEPES buffer pH 7.5 with 10 mM MgCl_2_ and contained 0.1 mM acceptor (compound **1**), 10 mM UDP-Gal*p*, 10 mM PEP, and 5 µM WbbY and 2.5 µM WbbZ. Reactions were performed for 15 min at 30 °C and stopped by addition of an equal volume of loading buffer before analysis by SDS-PAGE. The structures of products from reactions were predicted by mass spectrometry.

## Supplementary Material

Appendix 01 (PDF)

Dataset S01 (XLSX)

## Data Availability

All study data are included in the article and/or supporting information.
