# Supplementary material for: Identification of a second glycoform of the clinically prevalent O1 antigen from Klebsiella pneumoniae
Source: Proc Natl Acad Sci U S A. 2023 Jul 10;120(29):e2301302120. doi: 10.1073/pnas.2301302120 (PMC10629545; doi:10.1073/pnas.2301302120)
Supplement: Supplementary file 1 — Appendix 01 (PDF) [file pnas.2301302120.sapp.pdf]

SUPPLEMENTARY INFORMATION

A new epitope presented in a previously unrecognized second glycoform of the clinically prevalent O1 antigen from *Klebsiella pneumoniae*

Steven D. Kelly[1], Olga G. Ovchinnikova[1], Fabian Müller[2], Michael Steffen[2], Martin Braun[2], Ryan P. Sweeney[3], Michael Kowarik[2], Rainer Follador[2], Todd L. Lowary[3,4,5], Fabio Serventi[2§] and Chris Whitfield[1§]

[1]Department of Molecular and Cellular Biology, University of Guelph, Guelph, Ontario N1G 2W1, Canada
[2]LimmaTech Biologics AG, Grabenstrasse 3, 8952 Schlieren, Switzerland
[3] Department of Chemistry, University of Alberta, Edmonton, Alberta, Canada T6G 2G2
[4]Institute of Biological Chemistry, Academia Sinica, Academia Road, Section 2, #128, Nangang, Taipei, 11529, Taiwan
[5]Institute of Biochemical Sciences, National Taiwan University, Section 4, #1, Roosevelt Road., Taipei 10617, Taiwan

§ Co-corresponding authors.

Fabio Serventi. Email: fabio.serventi@lmtbio.com, Tel: +41 44 733 85 73
Chris Whitfield. Email: cwhitfie@uoguelph.ca, Tel: 1-519-824-4120 ext. 53361.

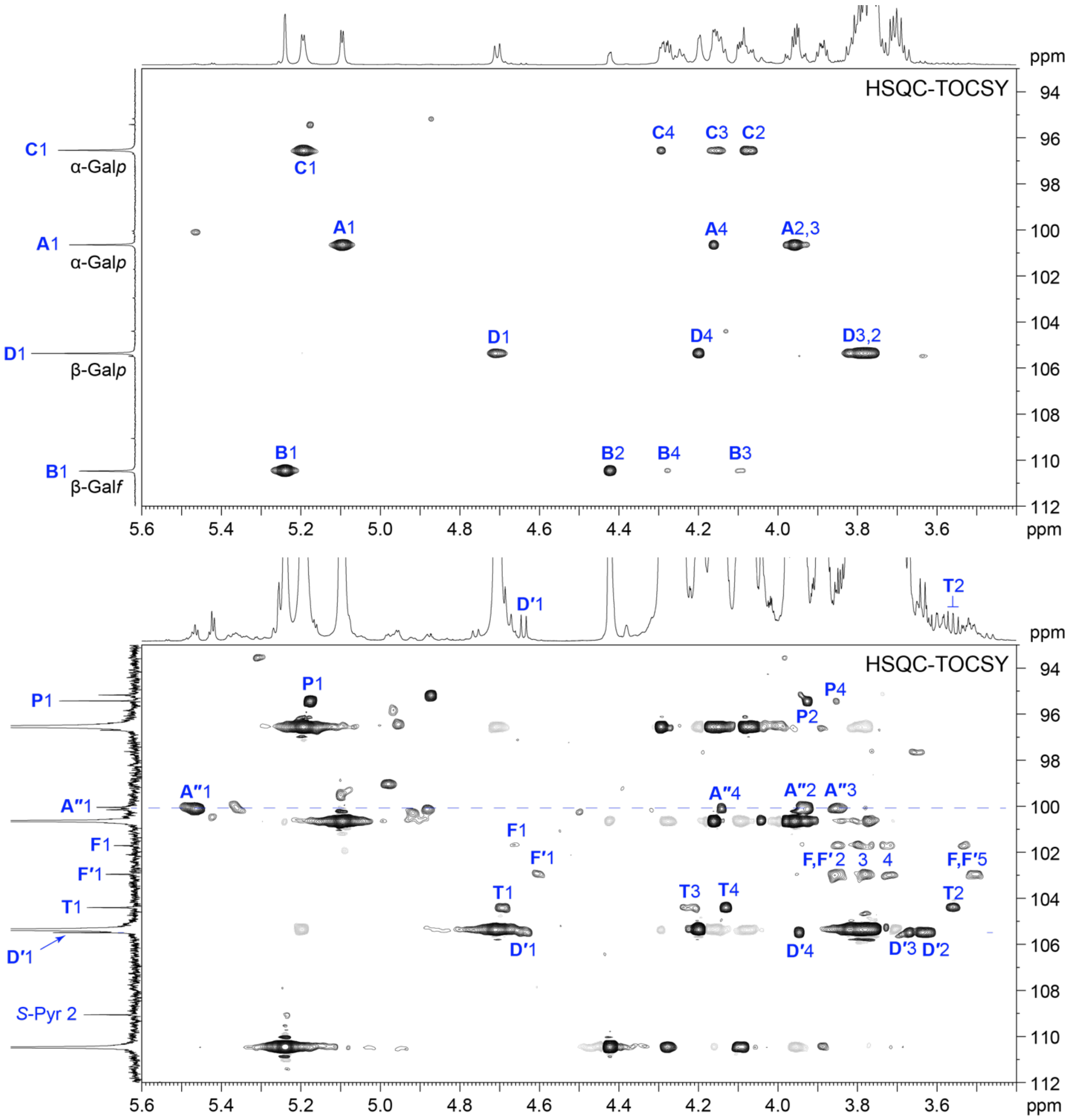

Figure S1. Parts of ^{1}H,^{13}C HSQC-TOCSY spectrum of *K. pneumoniae* O1b OPS. The upper spectrum (high level cut) shows the major correlations belonging to the internal repeat units. The lower spectrum (low level cut) also shows correlation from terminal residues. The corresponding parts of ^{1}H and ^{13}C NMR spectra are shown along the horizontal and vertical axes, respectively.

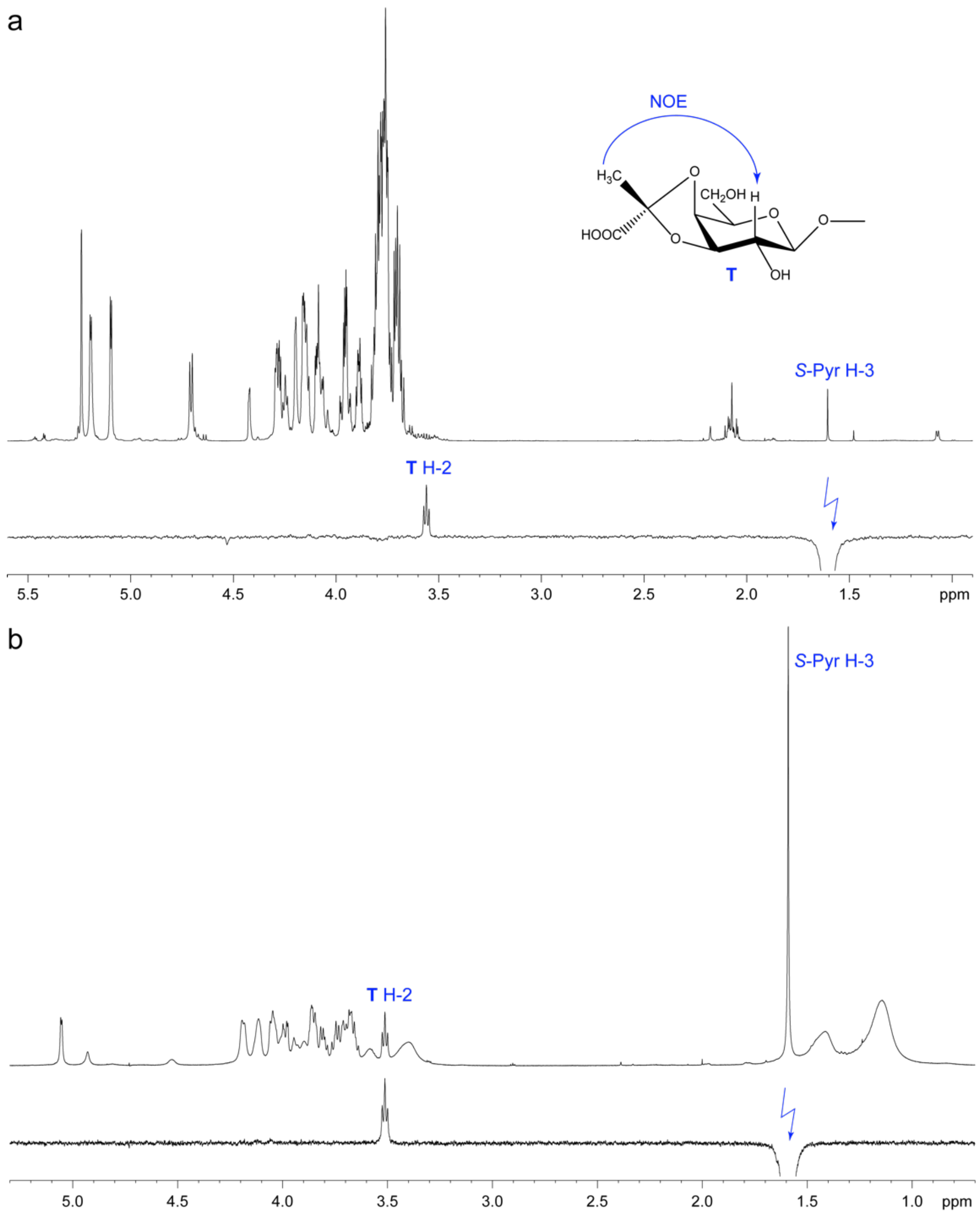

Figure S2. Selective 1D ROESY experiment. The experiment was performed with a spin-lock time of 200 ms and demonstrates the *S* configuration of the major Pyr residue in (**a**) OPS and (**b**) compound **3**. Selective irradiation of the Pyr methyl group signal at δ 1.61 (OPS) or 1.59 (**3**) results in the sole response from **T** H-2 at δ 3.56 (OPS) or 3.51 (**3**). In an *R* configuration, response from **T** H-4 would be expected instead (1).

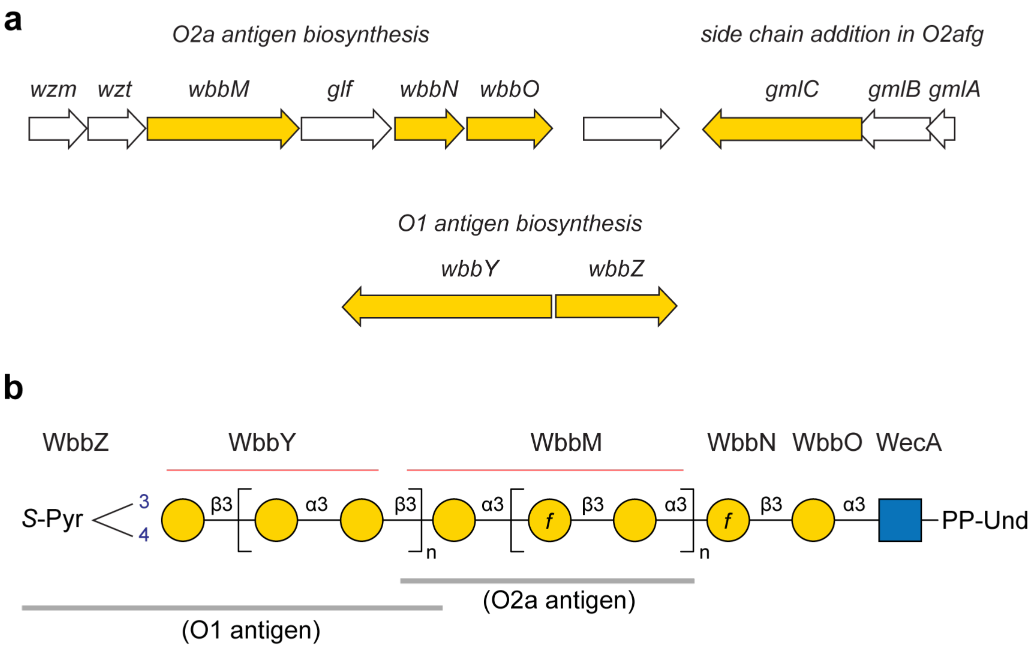

Figure S3. Biosynthesis of the "O2" and O1 antigens. **a**, genetic loci responsible for the synthesis of the O1 and "O2" antigens. The single ABC transporter encoded by *wzm* and *wzt* exports all of the O1 and "O2" polysaccharide products. **b**, The O-PS backbone is assembled in the cytoplasm as an undecaprenol diphosphate-linked glycan. All *K. pneumoniae* OPSs are initiated by the phosphoglycosyltransferase, WecA (from the enterobacterial common antigen synthesis pathway), which results in an N-acetylglucosamine (GlcNAc, blue square) residue at the reducing terminus of the OPS chains. The actions of each glycosyltransferase (GT) enzyme (yellow) are shown. WbbO and WbbN act on the product of WecA to create a trisaccharide acceptor for polymerization (2). WbbM (2) and WbbY (3) are polymerases for the O2 and O1 structures, respectively. The O2a backbone can be modified by the products of the *gml* locus in the periplasm to create the O2afg antigen (not shown here but refer to Figure 1). GmlA and B synthesize and export the undecaprenol-monophosphoryl-Gal*p* glycosylation donor and GmlC is the serotype-specific GT (4). Glf is a UDP-galactopyranose mutase that converts housekeeping UDP-Gal*p* to UDP-Gal*f*, to provide the two required donor substrates (5). WbbZ is the PEP-dependent pyruvyltransferase that modifies terminal β-(1→3)-Gal*p* residues to generate the O1b glycoform identified in this study.

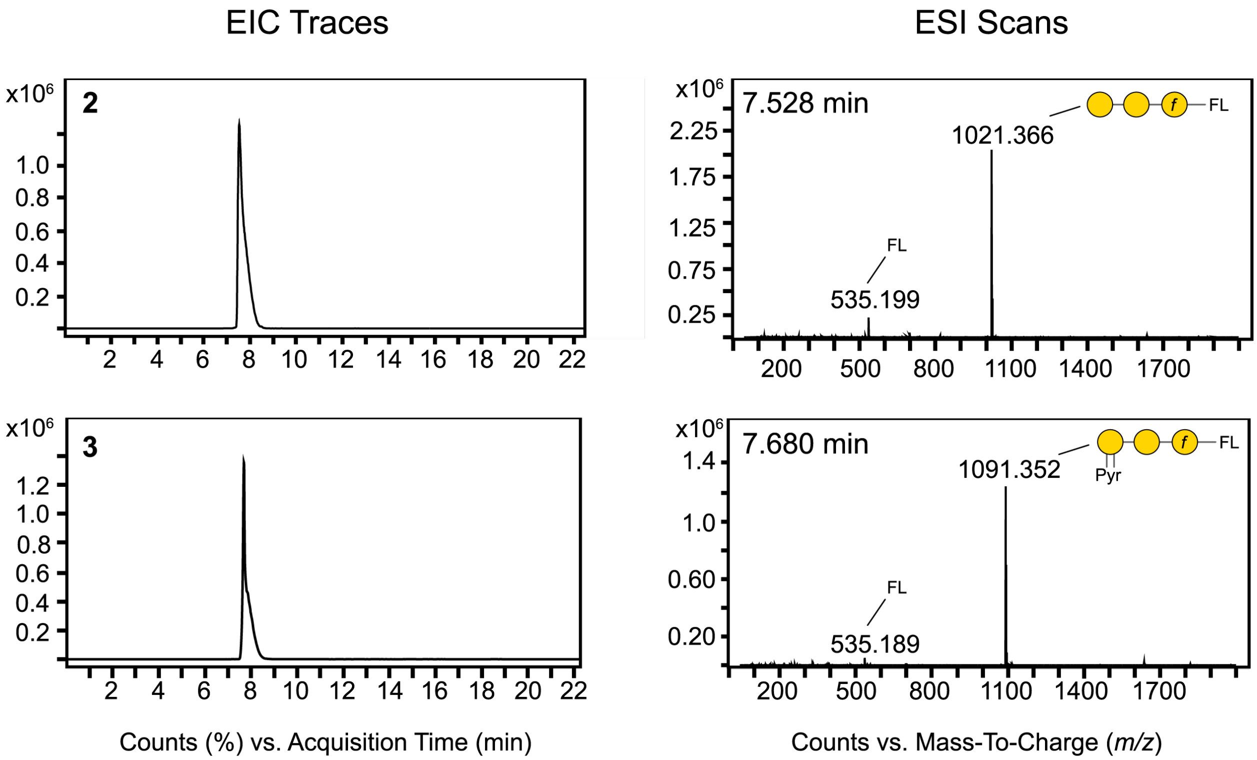

Figure S4. Mass spectrometry analysis of compounds 2 and 3. Extracted ion chromatograms (EICs) of *m/z* 1021.366 (**2**) and 1091.352 (**3**) are shown in the left panel. Shown beside the EICs are the ESI mass-spectra at the indicated retention times. ESI-MS was performed on the same purified compounds **2** and **3** that were analyzed by NMR.

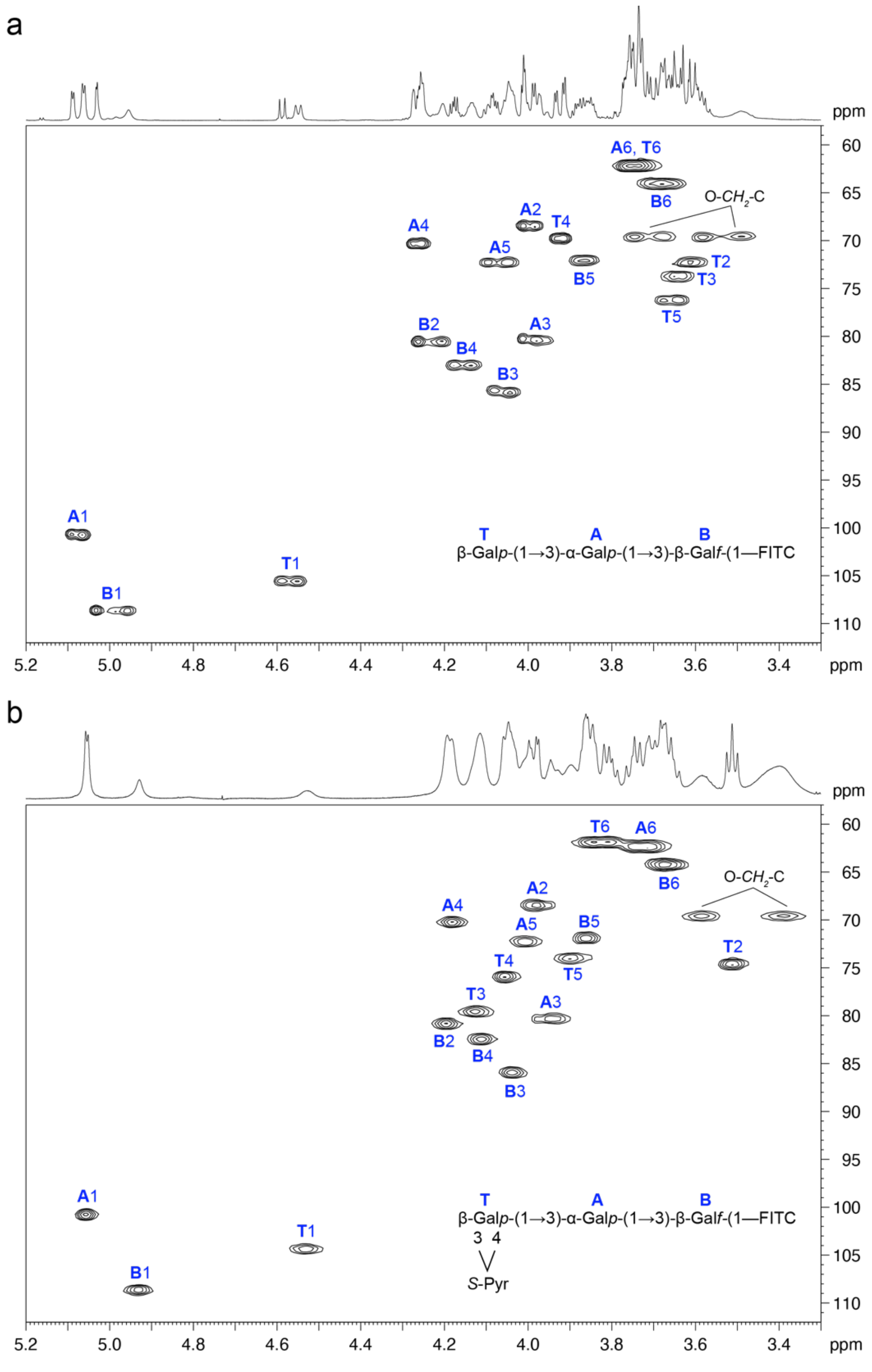

Figure S5. Parts of ^{1}H,^{13}C HSQC spectra for *in vitro* products. The spectra for compounds **2** (**a**) and **3** (**b**) are shown. The corresponding parts of ^{1}H NMR spectra are shown along the horizontal axes.

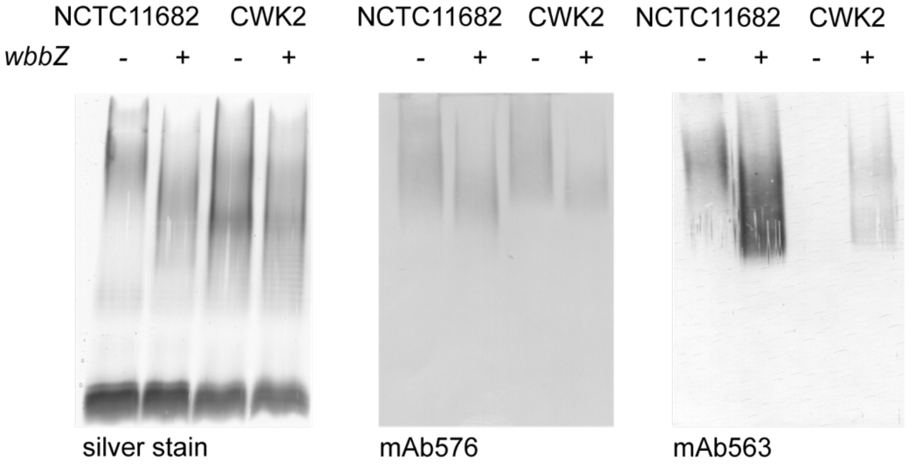

Figure S6. Transformation of *K. pneumoniae* CWK2 with a plasmid carrying *wbbZ* confers reactivity to mAb576. *K. pneumoniae* CWK2 (O1a) and NCTC11682 (O1ab) were transformed with plasmid pWQ1113 and the LPS molecules in whole cell lysates were separated by SDS-PAGE and probed in immunoblots with the indicated antibodies. Silver staining and western blotting was performed three times with the same results.

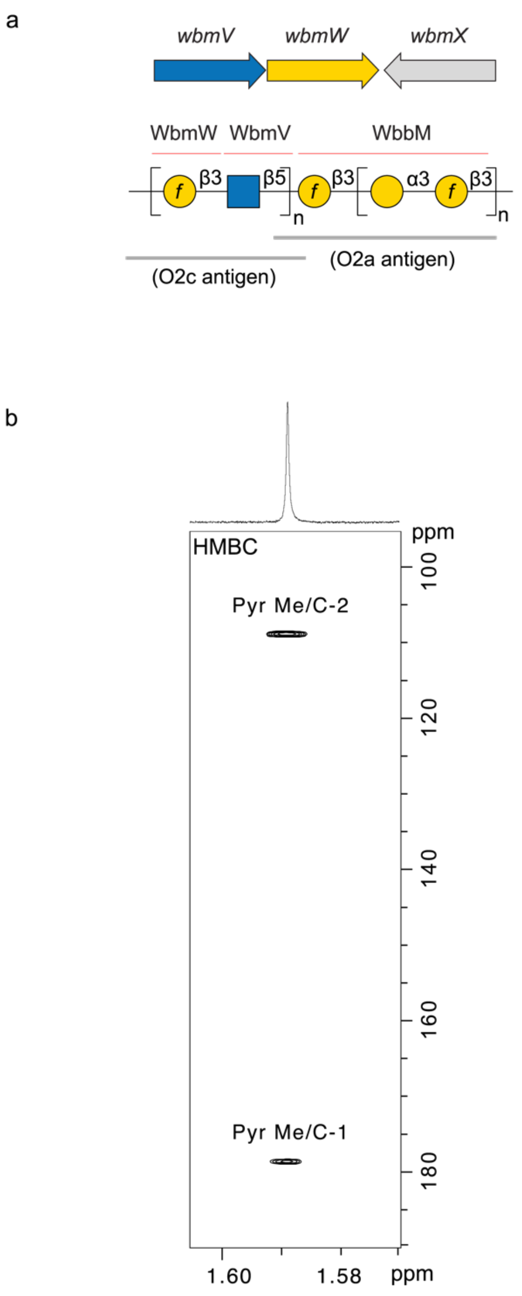

Figure S7. Identification of pyruvate modification in *K. pneumoniae* O2ac. a, The O2c modification requires *rfb*[2a] as well as a three gene locus *wbmVWX*. **b**, Part of the $^{1}H,^{13}C$ HMBC spectrum for the recombinant O2ac OPS showing correlations between the pyruvate methyl group and the acetal carbon (C-2) and the carboxyl carbon (C-1). The NMR data renalyzed here was published previously by some of the authors (4) but the presence of pyruvate was not recognized and this part of the spectrum was not presented in the earlier study

	1
Kp O1 WbbZ	MTNM..
Cv	MANA..
Ab K20	MNAK..
Ab K82	MNAD..
Pm O24	MINIKP..
Kp K13	MSHM..
Kp K26	MLAN..
Kp K30	MHQN..
Kp K69	MNQE..
Sd Type 9	MINN.......WV...
Kp K12	MKMK..NKVINK
Sp Pvg1p	MFANINIRKSVWLFLLAAVSCTLFIYGVTRTDLQTLKNPSSLTSPSSSTSVDKKKPLFTK
Kp WbmX	M...
Kp K5	MK..
Sd Type 10	MNK...
Pa CsaB	MASK..
Kp K11	M...
Kp K21	M...
Ep	M...
Ec K-12 WcaK	M...
Kp K32	M...
Kp K3	M...
Kp K68	M...
Kp K1	M...
Kp K58	M...
Sd Type 1	M...
Sd Type 2	M...
Ec O156	MNKK..
Pm O51	MI..
Pm O52	MINLNHI...
Kp K70	M...
Kp K80	M...
Kp K72	MR..
Sp Type 4	M...
Kp K6	MLNRII..KNK...
Kp K46	MFDFILSR..KSQ...
Xo	MANALLQK...WI...EHA...
Kp K14	MMETIKV...
Kp K64	MKEV.KV...
Kp K74	MKEV.KV...
Kp K7	M...
Kp K31	M...
Kp K56	M...
Kp K27	M...
Kp K36	M...
Rm	M...
Kp K8	MK..
Kp K33	MSKF..
Kp K35	MSKY..
Bf WcfO	M...

```
                                 10                  20              30          40
Kp O1 WbbZ    ........KLKFDLLLKSYHL........SHRFVYKANPG.....NAGDGVIAS.ATYDF
Cv            ........ELKFDLFLKSYHP........SHRFVYKANPG.....NAGDGVIAS.ATYDF
Ab K20        ......DLKRHLQSIIESKLSPL.....IDSNYVYWDLPYHI...NIGDTLIWQ.GTLDF
Ab K82        ......ELRLHLKSIIESNLTPL.....INSDYVYWDLPYHI...NIGDTLIWQ.GTLDY
Pm O24        NKYQNTSIKNYLTEIHNDNGK........FTSAIYLDIPCYH...NLGDMLIYL.GAKKM
Kp K13        .....IELKNNLNIILNAIPP........GSEIILLDTPLHL...NVGDVLIYK.GQLQF
Kp K26        ..KQMLNLKDHLSSIVKYIKN........KNNVVFLDYPLHH...NVGDLLIFQ.GTQQF
Kp K30        ......NLKNKLNEILPFIKD........KNEVIFLDYPVYL...NVGDLLIYH.GTENF
Kp K69        ......KLKIKLNEILPFIKS........KNDVIYLDYPVHL...NVGDLLIYH.GTENF
Sd Type 9     ..ALMDSLKNSHSEIANLI.........GEARIAFIDIPMYF...NVGDLLIYF.GTEAF
Kp K12        VKWKFINFSNKYYRLPNLDYT........QKIAYVLGVSNHP...NAGDQEITL.AQKKF
Sp Pvg1p      SPRNSASCESTITLQSNLLFTYYKHYFAGIKKVALIGFPDHP...NKGDSAIYV.AEKKL
Kp WbmX       ...............................KINLVGAVGGG...NFGDEFILNCCIAEH
Kp K5         .............................KNKVYIRGAYAPG...NIGDDVLML.SVINI
Sd Type 10    .............................KNEITLWGYYGFN...NLGDDVMLD.VCLKE
Pa CsaB       .............................VTRIVLSGYYGFN...NSGDEAVLL.SILTA
Kp K11        ...............................KLLLVGNHTCG...NRGDAAILR.GLLDT
Kp K21        ...............................KILLVGNHTCG...NRGDGAILR.GLVDS
Ep            ...............................KILLVGNHTCG...NRGDGAILR.GIIDS
Ec K-12 WcaK  ...............................KLLILGNHTCG...NRGDSAILR.GLLDA
Kp K32        ...............................KVLIINQHT..N..NFGDDAAGV.ALVST
Kp K3         ..............................KKLIIVNEVCSD...NIGDHAINL.GVLKI
Kp K68        ..............................KKVLAINECCSD...NIGDHAINH.GLQKL
Kp K1         ...............................FLFYKPTTQYE...NLGDALIAK.NLLDI
Kp K58        ...............................IFLYRPQTQYE...NLGDALIAK.NLLEI
Sd Type 1     ...............................IFLYRPQTQYE...NLGDALIAK.NLLEI
Sd Type 2     ...............................TTLLFGYFD....LNFGDDWL....IHEF
Ec O156       ............................DNFVNIFDTSIASL...NVGDYIIMD.AVTKQ
Pm O51        .............................NSIALYDPSIATS...NVGDEIISE.SVKNA
Pm O52        ............................NTKIGVLDTSICSM...NVGDFIIMD.SCYKV
Kp K70        .............................TKHVVLYGAFDRY...NYGDNLMPI.LLERF
Kp K80        .............................KYTIVLYGAFDRY...NYGDNLMPI.LFEMY
Kp K72        ...............................KYNIVGAFDRH...NYGDILFPL.IHSEF
Sp Type 4     ..................................LHGATDYGSS.NYGDYLYGE.IVYDL
Kp K6         .............................QKKNVFWWEPKDGS.HNAGDHLGKV.IVQSI
Kp K46        .............................EKKCVFWWEPKDGS.FNAGDHLSKI.VVQQM
Xo            .............................ERRALFWWQPKNAG.VNMGDHLSKV.IVSCV
Kp K14        .HWCYID......................................NFGDALNPY.LLEKL
Kp K64        .HWCNIE......................................NFGDALNPY.LISKI
Kp K74        .HWCNIE......................................NFGDALNPY.LISKI
Kp K7         ...............................KIYYFKDPV....GNFGDDLNSW.LWDTL
Kp K31        ...............................KLYYYKDVI....GNFGDDLNSW.LWDAL
Kp K56        ...............................QIYYYKDSV....GNFGDDLNGW.LWDRL
Kp K27        ...............................KVFYYKSKN....GNFGDDINGW.LWDRL
Kp K36        ...............................KLYYYESKT....GNFGDDINKW.LWDEL
Rm            ...............................KPYYWESQH....GNFGDDLNLW.LWDFL
Kp K8         ...............................NIGLFTSVYFN...NIGNAFIDF.GAQAT
Kp K33        .............................GVIVYEGGKFESNGILNLGDYVQST.AAKQF
Kp K35        .............................GVIVYEGGKFQEQNLLNLGDYVQST.AAKQF
Bf WcfO       .............................RKILLTYGDIKTI...NIGDYIQSI.AAKQF
```

```
                                                     50
Kp O1 WbbZ     FERNALT................................YIPYRDGE............
Cv             FERNALT................................HIPYRDDE............
Ab K20         LSDKPYK.................................MMDYGN.............
Ab K82         LSELDYK.................................MLDFGS.............
Pm O24         LHDLNVT.................................VRYTES.............
Kp K13         FKEHHIK.................................IISEHSDK...........
Kp K26         FADNDIH.................................VKFYRS.............
Kp K30         FKENNIN.................................VKLRRS.............
Kp K69         FAENDIK.................................IKLRRS.............
Sd Type 9      IKEYNLN.................................VIYRT..............
Kp K12         INKYFPN..............................YQYVEIEKE.............
Sp Pvg1p       LDALNIE..............................VVYITAQEAD............
Kp WbmX         IKNKDAKVSVSGFSPDIILKSG..................FSVENHSLNFFNILDKLRQ
Kp K5          VKKVVPE.............................KFISVGVEHPSLA..........
Sd Type 10     IKSTKKY....ETINVYGN....................YQEIDKIDGVVFHPLA....
Pa CsaB        LERAGEE...............................AGMAIEPVVLSGDPETT....
Kp K11         LRTLDAN....LEIDILSRYSKSSEYLLGETIIQDPLF..DKTIKKKAGALSTIINKAK
Kp K21         IKQINPD....VEVDILSRYARSSAFLLGTAIKQDVLY..SSRMNKNGGLVKKVIWKIK
Ep             LYLERND....LDIDVISRFPTSSSYLLQQDILPDELFLETKKSNS.......LVTKIK
Ec K-12 WcaK   INILNPH....AEVDVMSRYPVSSSWLLNRPVMGDPLFLQMKQHNSAAGVVGRVKKVLR
Kp K32         LLKSKHC....VDVTYIWNKNGTK................IPIEDAALTHYAE..KNLS
Kp K3          LDEYNFKGISYGFDAEKKNVGP..............TIKKSKKESSFIRFLKFIKNKTL
Kp K68         IMINSYEAQSVGFDAGVKHEKP................QKIATDNVHLSFRTRFKKKYL
Kp K1          LSKKGTI................................FLLTEDIPKGFVDIIKNDNC
Kp K58         LENYGEI................................YIDDKNVPSNYIEIIKNKAC
Sd Type 1      LENYGEI................................YIDDKNVPSNYIEIIKNKAC
Sd Type 2      INQYHKD............................NVILVVSDENLYLPFKHDIR....
Ec O156        LSTISNL............................PQTVTLPTHD..............
Pm O51         LTDIFNY............................SQIITFPTHS..............
Pm O52         VSDLFPN............................AQKIHFPTHE..............
Kp K70         FLSEYVK........................CSKDYEFIYASINSSDLSC.........
Kp K80         MVKNHPD........................IEQRANFIYASIQDSDLSC.........
Kp K72         IRRHSES............................AEINYYSLSESNLESIGGVR....
Sp Type 4      LESKGYE.................................ASFYNPSDFFQMYLK....
Kp K6          LSTRDFE....................................................
Kp K46         LALKDLE....................................................
Xo             LSLQDKT....................................................
Kp K14         TGKKVVY....................................................
Kp K64         SGLPVKY....................................................
Kp K74         SGLPVKY....................................................
Kp K7          IPGYFDS....................................................
Kp K31         IPDFFDQ....................................................
Kp K56         LPNF.......................................................
Kp K27         LPNFFDN....................................................
Kp K36         KPGFFDC....................................................
Rm             LPGFREV....................................................
Kp K8          IEGALPDG...................................................
Kp K33         LPYVNK.....................................................
Kp K35         LPSVDH.....................................................
Bf WcfO        FDDDNYI....................................................
```

```
                                                         60
Kp O1 WbbZ    .........................................RYS.......SETDILIFG
Cv            .........................................RYN.......SDTDILIFG
Ab K20        .....................SATASLTP....LD.................SNVVILLH
Ab K82        .....................YGTIAFPEI.....................DKNIVILMH
Pm O24        .....................ALSHNLKKIKRILKQ...............HPDTVILMQ
Kp K13        .....................ASKSFIVKNHRKIP.................RNAIIVLT
Kp K26        .....................VLDYNVDELKKSID.................AETTLICH
Kp K30        .....................NARYSVKEIEKYLN.................PNTVILMQ
Kp K69        .....................VAKYSVADIKRKIT.................PNTTILFH
Sd Type 9     .....................GMNVDLKKLK.....................DVDVILFQ
Kp K12        .....................KTIFIISELRKRLK.................PDDIVFIQ
Sp Pvg1p      ........................YSASELKSIISDIP.............RDEFALAFH
Kp WbmX       KAEN.................GLKISMNDLISQFEGA..............EEFDAIHFI
Kp K5         ..................RKYNSNIDWIHIKKPIS..................ADVVVFG
Sd Type 10    .....................GIKNKVEFLKSILK..................SDKLIWG
Pa CsaB       ..................TRLYGVQAVHRMKPGALLGAI............RSSDGLISG
Kp K11        NKCLPLVLISHIKKKGVLKAFPLPQHIREFTQKLK.................EYDAVIQV
Kp K21        NSILPDILVSHAKGKGFLKYFPLPNNVVEFIKDLE.................KYDAVIQV
Ep            RRLMPKIMMAHIRGSGFFKNFAVPEYLQKFTDKLK.................QYDAVIQV
Ec K-12 WcaK  RRYQHQVLLSRVTDTGKLRNIAIAQGFTDFVRLLS.................GYDAIIQV
Kp K32        RANLLKQLFAFI.........FGNDSYFHLIKKLSM...............NADYVLVSP
Kp K3         KNNRFYK...........YSLWILRNYSRVSSITKQ...............NKGQAIIIG
Kp K68        SGRQSLK...........YALWLIKNLLRVRSTVK................NEHQAAIIG
Kp K1         VGIS.................KVKFALMPLVYRLL................NRDVAFIYK
Kp K58        VSHEV...............YKCHFAFLPIKLRLR................GKAVCFVFK
Sd Type 1     VSHEV...............YKCHFAFLPIKLRLR................GKAVCFVFK
Sd Type 2     ....................FRKVSDLGKIKAFLQ..................SDEICIV
Ec O156       .....................HFGREGRRLLSLAK.....................YSIV
Pm O51        ...........................RRSLNIIKK...............ANSCDLSFV
Pm O52        ...........................RITRVGLKR...............QKEIELNFL
Kp K70        .....................YKCKPTVPIKSLLKN...............DIDYNIIVV
Kp K80        .....................YGCKPTIAMNRLLDI...............PSGSTVVVV
Kp K72        ..........................TKSIKSLLNKSVRS...........SPEDRVIMC
Sp Type 4     .........................EYRQKQSFTK................KQADAILYI
Kp K6         ...............................................ILGKKSSHNTLFS
Kp K46        ...............................................LTQKNNLKNKLMA
Xo            ...............................................LIEKQDLSKKLIA
Kp K14        ..................RNFDKPNYKKEIKLLIKSLLQFKSYDFNRLRAFDENENVVLG
Kp K64        ..................RNYKTPSYYKELRSLAKCILTFKKYDFNRMLPYNRRESVVLG
Kp K74        ..................RNYKTPSYYKELRSLAKCILTFKKYDFNRMLPYNRRESVVLG
Kp K7         ....................................................DDTVRLSG
Kp K31        ....................................................DESIRMSG
Kp K56        ..........................................................SV
Kp K27        ....................................................DENTRLSG
Kp K36        ....................................................NKDIRFSS
Rm            ....................................................YPETLLVG
Kp K8         ..................YQLVKVSQFQSFVNAMQAGMGL...........RESRVVRAV
Kp K33        ............................................................
Kp K35        ............................................................
Bf WcfO       ............................................................
```

```
            70                               80
Kp O1 WbbZ   GGGNLIE......................G.LYSEGHDFIQ...................
Cv           GGGNLIE......................G.LYSEGRDFIQ...................
Ab K20       GGG.NFG......................D.IYGSSQKFRK...................
Ab K82       GGG.NFG......................D.IYTTSQDLRK...................
Pm O24       GGG.NFG......................D.LYSEAQTYRL...................
Kp K13       GGG.NFG......................D.LYQHHQTLRE...................
Kp K26       GGG.NFG......................D.LYDVHQNLRI...................
Kp K30       GGG.NFG......................D.LYPEFQKFRQ...................
Kp K69       GGG.NFG......................D.LYPEYQKFRQ...................
Sd Type 9    GGG.NFG......................D.LYPHHQQLRE...................
Kp K12       GGG.TLS......................D.LYPEHEVPRQ...................
Sp Pvg1p     GGG.NFG......................D.LYPDHQHLRE...................
Kp WbmX      GGG.YIN......................S.LWPSNYALLG...................
Kp K5        GGGQFFSFNPPPGVKSSLISKVIKSIKAQENLFTAIERLMV...................
Sd Type 10   GGTCFYK......................DNNTGYSGLFLL...................
Pa CsaB      GGS.LLQ......................D.ATSSKTIPYYLGV................
Kp K11       GGS.FFV......................D.LYGVSQFEHI...................
Kp K21       GGS.FFV......................D.LYGTGQFEHI...................
Ep           GGS.FFV......................D.LYGPLQFEHS...................
Ec K-12 WcaK  GGS.FFV......................D.LYGVPQFEHA...................
Kp K32       GGA.NIG........................IYKDWAYLVN...................
Kp K3        GGQ.LIQ......................S.........................GG...
Kp K68       GGQ.LIQ......................S.........................GG...
Kp K1        PGH.LFG......................G.SKEYGTGIKA...................
Kp K58       PGH.FFG......................D.TKSLGGWKRI...................
Sd Type 1    PGH.FFG......................D.TKSLGGWKRI...................
Sd Type 2    GGS.MFQ......................DSKHWFGHYLKL...................
Ec O156      GGTNLLS......................SHVTQYRQWRFR...................
Pm O51       GGSNLLS......................PKMLRYQQFKLS...................
Pm O52       CGTNCLN......................SSMFLHRQWNVG...................
Kp K70       GGE.VMGADV...................GTLFSHVQNNIFYAKAI..............
Kp K80       GGE.VLGANV...................GTLYTHVQNSLLVTQAL..............
Kp K72       GGD.ILTVDWVGML...............GHLSGENFYFLL...................
Sp Type 4    PGG.YFGEGHN..............ARFRDNLIQFKRFLPL...................
Kp K6        IGS.VMHFAK...................N..............................
Kp K46       IGS.VMHFAN...................E..............................
Xo           IGS.VLHFAK...................D..............................
Kp K14       IGS.LLNRSR...................P..............................
Kp K64       IGS.LLDRSR...................S..............................
Kp K74       IGS.LLDRSR...................S..............................
Kp K7        IGT.IINTSMP............MA....R..............................
Kp K31       IGT.IINTAMP............KA....D..............................
Kp K56       IGT.IINEVMP............KA....D..............................
Kp K27       IGT.IIDSYMP............HA....R..............................
Kp K36       IGT.IINKGMP............AA....K..............................
Rm           VGT.VLNRALLP...........KA....T..............................
Kp K8        WNG.VMKNHAH...............KIHDKLYTAIPTKKTITLMDYVKLDALIIPGCVL
Kp K33       ....YVT.....................REGMHLYSGEHIK...................
Kp K35       ....YIT.....................REGMHRYNDETVK...................
Bf WcfO      ....FFN.....................RDELKLYKGEPAK...................
```

```
                          90                                         100
                          .                                          .
Kp O1 WbbZ      .........NNIGKFH...........................KVIIMPSTIRGYSDL..
Cv              .........NNINKFH...........................KVIIMPSTIRGYSDL..
Ab K20          .........DVIELYPDN.........................KIIILPQTIYFKNKDVE
Ab K82          .........DVVEKYKEN.........................KIIILPQTIHFYDKEKE
Pm O24          .........NIIKKFKNE.........................KIIILPQTIWYDNYNNY
Kp K13          .........TVCKYLPNN.........................KIVQLPQTAHFENQSKL
Kp K26          .........SLVENFKEN.........................RIIIFPQTAHFSDLDKL
Kp K30          .........DVVSSFPDT.........................RIIILPQTAFFENEHEL
Kp K69          .........DVVKSFPNN.........................RIIILPQTAYFESENAL
Sd Type 9       .........KITLKFPNK.........................KIICLPQTLHFNNEEEL
Kp K12          .........ILLKELKDS.......................PCKIVQFPVSFYYEDFDKF
Sp Pvg1p        .........LVVRDFPSF.........................TTISFPQSVW.YNEQQL
Kp WbmX         .......IAYIYSKIHKK.........................PIYATGLGLSPYEENE.
Kp K5           .....SSCGGLDKIYIPR.........................KLAAFCIGLGPFDNHGK
Sd Type 10      ......FVISLFCVLFRK.........................KNFFLGIGLGKLNSSLS
Pa CsaB         .........LKLAQWFRK.........................PTFIYSQGVGPVNRQSF
Kp K11          ..........LCALLANK.........................KIYLIGHSVGPFEGKNF
Kp K21          ..........LCSNIAGK.........................PIYLVGHSVGPFENPKF
Ep              ..........LCALLAKK.........................PVYMIGHSVGPFQKERF
Ec K-12 WcaK     ..........LCTFMAKK.........................PLFMIGHSVGPFQDEQF
Kp K32          .........VIIARKYNK.........................NVVFHLNTISKSNSILF
Kp K3           TFAIAMYTWTLFSKLRNV.........................DIYIIGVGCAEHFDRID
Kp K68          TFPIAMFVWTFYCKKKNL.........................PIYIAGVGCAEKFSKLD
Kp K1           .........LLKISYVNVLHFI...................GVKIIKTGVSVGPLSGSFL
Kp K58          .........MLMTAYLTVLKIF...................GVKFLRTGVSVGPLNSGYM
Sd Type 1       .........MLMTAYLTVLKIF...................GVKFLRTGVSVGPLNSGYM
Sd Type 2       ......YIYLLIARILGK.........................KNYVIGCNLNAIKNKKL
Ec O156         ..........MTDLLFLK.........................KCILMGVGWWQYQDKPD
Pm O51          ..........IYDFIKTK.........................NLITLGVGWQYYQDKID
Pm O52          ..........YLNSILMK.........................PIVSLGVGWGNYQNAPD
Kp K70          .....NFTKRVSPKFVNAYAKLFYPAAWDYPYIPRKNSFKGNVKIIYNTVGGVPVKSQWE
Kp K80          .....RFIRKLSPSLLNRIALRRYKAVWEYPYIPDIKDFENKIKVIYNTVGGNPINAQRE
Kp K72          ....KVARKLLGVSLSNQIIRIIWGQKNKYPYIITHKNI..ECKIFYTGVGGSGFELSKS
Sp Type 4       .........GIWASYFKK.........................PIGVLGIGAGPNNDSLM
Kp K6           ...........................................GDCIWGTGINGKID...
Kp K46          ...........................................NDCVWGTGINGKIS...
Xo              ...........................................GDTVWGSGINGKIP...
Kp K14          ...........................................RFHIWGTGYMNQHERAK
Kp K64          ...........................................NFSVWGSGYMNNFERAE
Kp K74          ...........................................NFSVWGSGYMNNFERAE
Kp K7           ...........................................KWFVFSSGVGYG.....
Kp K31          ...........................................KWVVFSSGVGYG.....
Kp K56          ...........................................KWFVLTSGVGYG.....
Kp K27          ...........................................KWYVLSSGVGYG.....
Kp K36          ...........................................KWIVFSSGIGYG.....
Rm              ...........................................HKLVIGSGFGYG.....
Kp K8           TVPFFKIFGAELEALKNK.......................GTELIFLGASGNYYTDYEV
Kp K33          .........MIMNAWYMA................NPENFPPSKDIEPLYVSVHLNSSIVD
Kp K35          .........MIMNAWYMA................NPENFPPSPKIDPLYVSVHLNSSIVD
Bf WcfO         .........VIMNAWMTY................KPYNWPPSSQVYPLFVALHINSSAES
```

```
                      110        120        130                     140
Kp O1 WbbZ     .........FINNIDKFVVFCRENITFDYIK......SLNYEPN............KNVF
Cv             .........FTNNIDKLIVFCRENTTFDYIK......SLNYEKN............KNVF
Ab K20         K....EDFQYFAQHKNLYLCVRDQASYDLACR......YLEV.............DKVL
Ab K82         K....EDLIYFSKHKNLFLCVRDKLSYEIAIR......YLDK.............SKVL
Pm O24         L....NDKEIIKKAKNLTIYTRDERSFNYISN.......ITS.............IKIN
Kp K13         L....ESSKIFRTHKDIVMFARDDSTFEIFSNYFS....................DNVY
Kp K26         N....HDISIFRKHSDVILFARDAKTFDLFQR.FS....................DKVF
Kp K30         D....KSVKAFGAHKDLIIFSRDTRSLSIFKK.FT....................SQTF
Kp K69         E....SSVKEFGEHKDLIIFSRDTRSIPIFNK.FT....................PNTY
Sd Type 9      E....RSAKIFKKHSDFHLYVRDQESLTIGYQ.FT....................DNIK
Kp K12         R....ASKSIYNSVKNLTIFARESKSLSVLKSELS....................VPVL
Sp Pvg1p       LE...QASILYAENPNITLVTRDRQSYGFAVD.......AFGKH............NEVL
Kp WbmX        .....SLTSLFNSLD..IIDVRDEKSKSLIP......SASFTGD............DALL
Kp K5          GYQ..RAVEIINKCD..YVSVRDDKSKSYCKMLGN....................ESVS
Sd Type 10    RV...LTITTLSLST..LSIFRDVNSYNKALSLVNP...................KKVG
Pa CsaB       YP...YIRHVFSRSA..YVSVRDRESAELLM......RMGVGQ............DAIQ
Kp K11         NR...VAKYSFSHVE..LVQLRERVSYDIMV......KYGFDM............KNVS
Kp K21         QH...IAKYCFTKAE..KIILREDVSLSLME......KDNFNL............NNVT
Ep             NQ...IANFVFSRVN..SLVLRESVSLEMME......KAGITT............QKVI
Ec K-12 WcaK   NQ...LANYVFGHCD..ALILRESVSFDLMK......RSNITT............AKVE
Kp K32         NL...IAKCVLKKC...VMFVREKASYDLLR......SQGIVS...............T
Kp K3          RF...LFKRSLSRAK..DILVREKSSISKIKDFFD....................CDVR
Kp K68         VW...LYKVSFKRAK..KIFVRDVSSRDKLKSIFS....................TESE
Kp K1          .....KAEMNISAKSY.IYGVREDYSLKFVE......GNSF...............KKYK
Kp K58         .....LYERFLNKIIN.FTGVRENKSIKYLA......ENGIT..............KNIK
Sd Type 1      .....LYERFLNKIIN.FTGVRENKSIKYLA......ENGIT..............KNIK
Sd Type 2      NM...LLKLLYKQVD..HFRVRDSNSVKILNK.....DYNINE.............ALIS
Ec O156        RVTKFILKHILHNEM..LHSVRDSFTLKQLH......SIGI...............TNVI
Pm O51         FLAQYFYRNNLSKDY..ILSVRDDYTMERLN......SIGI...............KNII
Pm O52         LYTKILLKKIFSNQH..LLSVRDSYTEDKFK......LAGL...............KNVI
Kp K70         YV........KDAD..YVSVRDQRSYDGMKKI.......................CNLN
Kp K80         NV.......ISAT..YVSARDNRTYNNLKEF.......................SQPV
Kp K72         KFIH.DIPRELIYVD..SISVRESITKEYLV......RGG................VSCN
Sp Type 4      NY...GIKRIINHAQ..FITVRDRESFDSLKHLSP....................SAPVH
Kp K6          .....KSKLKFNSLD..VRAVRGPNTRDFLK.....KEKGI...............IAPE
Kp K46         .....LDALKFKNLD..VRAVRGPKTRKLLL......DMGL...............QVPE
Xo             .....AERNTFSTLD..VRAVRGPKTRKFLL......DRGI...............AVPE
Kp K14         GGL.............LYAVRGRYSAEKLH......SEGF................EYCD
Kp K64         GGT.............LHAVRGRFSAEKLM......KEGF................PYCS
Kp K74         GGT.............LHAVRGRFSAEKLM......KEGF................PYCS
Kp K7          .....YPPVNFGDDNWNILCVRGPLSAKILGL.......................DKNK
Kp K31         .....NPPDNFGDGSWDILSVRGPLSAKVLGL.......................PKDK
Kp K56         .....HLPEGFGNDNWKILSVRGPLSAKVLGL.......................GSDK
Kp K27         .....FPPSHFGKDNWNILCVRGPLSANILNL.......................PPEK
Kp K36         .....YAPKGFGNESWDVICVRGPLSASILGL.......................PDNK
Rm             .....TLPDMSDPKEWDIRSVRGPLTAAKVGV.......................APEL
Kp K8          S....FVKEYIKKLEPKGIIFRDHVAYENYKDIVEVSHNGI...................
Kp K33         KIFRPEVIEHFKNNQ..PIGCRDNHTRDLLR......QKGIDAYYSGCMTLTLGYTYKRE
Kp K35         KIFRPEVIEHFRKHE..PIGCRDNHTRDLLL......AKGIDAYYSGCMTLTLGHSYKRT
Bf WcfO        RFLSHDSIKYLKKYE..PIGCRDYHTMNILK......GKGVNAYFSGCLTTTLGKTYKYN
```

```
                      150                           160       170       180
Kp O1 WbbZ    ITDDMAFYLDLN....................KYLSLKPIYKKQANCFRTDSESLTGDYK
Cv            ITDDMAFYLDLS....................KYLSLKPVYKKQVNCFRTDSESLTGDYK
Ab K20        FLPDMAFCIKDE.IL...............V.....KSKPTGKNLLMSRIDVEAVPVD..
Ab K82        LLPDMAFCIKPE.RL...............I..SQKKAPTSSRSLLMRRIDVEAKKVD..
Pm O24        MMPDTAHWLWDDGIN...............ILNKK.INHKGTKKILIHRTDDELSTESLK
Kp K13        KCPDMAHALYGI.YP..................KQTKEMINNNVMYMIRNDKEGSQQD..
Kp K26        LMPDMAHQLYGN.LP...............I.....CNVENNEALYFLRVDKEKNDLQLT
Kp K30        LMPDMAHSLWGT.LP.....................KSQKSKGTLYLIRNDKEINKVQRR
Kp K69        LMPDMAHSLWGT.LP.....................KSTHQTGALYLIRNDKEINEVQSK
Sd Type 9     LIPDMAHSLHPL.ID...............I.NECDINRESYRILHMVRSDKEANSNNAN
Kp K12        HVPDIVLSQDES.......................SNQPRNNDVVIMFRSDKECVLPQSL
Sp Pvg1p      LTPDIVFFMGPI...................PEIREATPITHDVLILARLDHEGGQQ...
Kp WbmX       ALNEPSMLVKND............................DSPALILSLQSHL.......
Kp K5         EFTDPTLLSDIW.YP..................NQIKKPYYSDKGYISIVLRD.......
Sd Type 10    FGGDLVLLYDIL.....................ERSNKFYGIERRYISVSGHYAYANDVG
Pa CsaB       VVPDPVMGLRLP......SSKPGQP......ALDHGKGFDASGRPYVGVSLRF.......
Kp K11        LSVDTAFLVGQNPDE.SNNYMIDH............WSKLISDKKTIAITVRKLAPFD..
Kp K21        LGVDTAFLVDTE.KD.VNDYAVSH............WKKIIKEQKTVALTVRKLAPFD..
Ep            PGADTAFLVRTRTLD.APGHNLIY............WQKQIAATKTIAITVRELAPFD..
Ec K-12 WcaK  HGVDTAWLVDHHTEDFTASYAVQH............WLDVAAQQKTVAITLRELAPFD..
Kp K32        LGVDTAFLLDKR.......................SKSERYEGKKILTFVPTELSNWHVH
Kp K3         YIPDLAYALFKQ......................DDYNHKKDNLAIIGCTAYYVYMKNIN
Kp K68        LIPDLAYALYDE......................GTFFSDKKERTLIGCTSYYVYEKNIK
Kp K1         RVSDLAYYSILK......................NREKYLDEKRDTISYSFRPYKQSNLP
Kp K58        KVKDLAFWSLNK.......................EPLKLNENQKYIAYSFRSF.NSNID
Sd Type 1     KVKDLAFWSLNK.......................EPLKLNENQKYIAYSFRSF.NSNID
Sd Type 2     VKPDLADKSKLS........................LTVTKENSCAVSIINNK.......
Ec O156       NTGCPTMWDLTP....................EHCCNIPREKGRRVL.............
Pm O51        NTGCPTMWKLTE....................EHCKKIEQHKKQNVI.............
Pm O52        NTSCSTMWTLTP....................EHCELIPKNKANDVV.............
Kp K70        LIPDSVLIASKL.VD..........NDFFKSNVRPGIIALCQNKRFITL...QACPYKVK
Kp K80        LVPDSVLMASTI.ID..........DSFFDSVVRHEVRQIVAND.FISI...QACPYKVN
Kp K72        LLPDTALIMSDY.YP...ISKLAE....VKWKNNVVSSNNFSFENYIVFQCAR.......
Sp Type 4     ETFDLIISSKLR......EEKTEQ.........LCQLKREAKDKKIILV...........
Kp K6         VFGDPGLLLPLF................................................
Kp K46        VYGDPGLLLPFF................................................
Xo            VYGDPGLLTPMF................................................
Kp K14        VWGDPALLLPIV................................................
Kp K64        VWGDPGLLLPRV................................................
Kp K74        VWGDPGLLLPRV................................................
Kp K7         AITDGAALLNTL................................................
Kp K31        YITDGAALLNTL................................................
Kp K56        YITDGAALLNTL................................................
Kp K27        FITDGAAFLNKI................................................
Kp K36        YITDGAALLSTL................................................
Rm            GIIDPAVMVADL................................................
Kp K8         ...DNAFFVNKVTVP.K.....................NIASEDYVVLNFDHP.......
Kp K33        KISDEVIFTDIM.............................HDSLSIKQLIKQPLRLG..
Kp K35        KVTDEVIFVDIM.............................HDSLSMKQLIKQPLRLG..
Bf WcfO       GKREGIYIVDPL.......................SYMPNGNNFFEIMKAVVQTVFYM..
```

```
                              190
                                .
Kp O1 WbbZ     .........ENNHDISLTW.........................................
Cv             .........ENNHDISLTW.........................................
Ab K20         .....KKY.IDQVDLQSDW...PTFEKSPIY..CIYLRLLLALNRKVSNGLQDKSSSYLV
Ab K82         .....DNL.YKLTDVHSDW...PTFENIPYS..MLTIMRLCNLSKKILKS.KNGNKTFLS
Pm O24         .........DDNYHKIFDW...PNLESSKDK...LFEKIIRF..........QQRKLPFL
Kp K13         .....K...NYASCVKEDW...DTICNINDK...NKLKKLLFLEKL.....NRATGLNLF
Kp K26         YA.......NNNEIRSVDW...VDFINPYEF...RYEFLLKKISRI.....ANKYNSSTM
Kp K30         L...LEH..KKGDDKYVDW...EDILTSKDL...LMRKLCRKLDGI.....GGVTNLSVL
Kp K69         V...LK...RNGDKQIVDW...ENILTRKDM...YMRVFCRKLDGL.....GSAVNLPIL
Sd Type 9      .........NMLSKRSFDW...ANIISPSMY...LMGRSIIILDKL.....RYSNSMKF.
Kp K12         .....................ANSIVDHFSK......................SNNIILT
Sp Pvg1p       .........HGAEDYYRDTLNAANLTYSVEDW............................
Kp WbmX        .........FEGQSLIEKIFTDGTFKELKNK....KIKKIIIIEAA......PEDNIPFS
Kp K5          .........WPHDKNGQDF..TKELIKFGNYL.INNGEKVRFVSVYKER....EEDLIAK
Sd Type 10     Y...IENF.............TCAIIKIAKH...HNIKDIVFVSMHQMP...ESNDHIFH
Pa CsaB        .........WNQDRSDMDAI.ADMLLQLSRM....RKVHLRFLPFHGAS..DEEASRYVM
Kp K11         .....KRLGVTQDQYESAF...AKIIDHYID....LGYQIIAFSTCTGIESYNNDDRIVG
Kp K21         .....KRLGVSQDEYEASI...ARIVDYLNQ....LGYQVIIFSTCTGIESYHSDDRMVA
Ep             .....KRLGVTQQEYEMAF...GKVINAMID....RGYQVVALSTCTGIDSYHRDDRMVA
Ec K-12 WcaK    .....KRLGTTQQAYEKAF...AGVVNRILD....EGYQVIALSTCTGIDSYNKDDRMVA
Kp K32         F....KN..INDHDLLANKI.VPSISKFAKN....DGYIVKILPHLYAS....EAEGKFL
Kp K3          E.LGLKDI.MTFEQYINAW...ISIILDECN.....EHRVLLLSTT.......VEDAEFS
Kp K68         E.LSRKDG.LTNSQYIDKW...QSIVLSELN....NKKEVILASTT.......VQDAVLN
Kp K1          PELQAKKI.............ASAILDVATKN......SIKIVNNVTQV....QTDIVFN
Kp K58         DKM.VKRI.............AVILEKMHEKY.KSDGISCKLISVT.....QVQRDLQFN
Sd Type 1      DKM.VKRI.............AVILEKMHEKY.KSDGISCKLISVT.....QVQRDLQFN
Sd Type 2      .........NTGKKKYYDWL.TSNFKELRRK..GIYHFKLFGFDSGAESDEKSINDFLVL
Ec O156        .........FTLTDYNQDLSADALLINTLKR....HYDEVLFWPQG.......SEDIHYM
Pm O51         .........FTLTDYSQNYELDRKLIEILKR....NYKNIYFWPQG.......LKDMEYL
Pm O52         .........FTLTDYRKNKEFDLTLIRALKS....TYKNVYFWAQG.......SQDYNYF
Kp K70         .........FTPKDM......ALVLDDIKRE....KAMDVILLPIGYAS...GHDDSLFL
Kp K80         .........FSATQL......ASELETVSSK....YSLKVVLLPIGYAS...GHDDVVFL
Kp K72         .........ANGEGYEDQI..VNEIIEINKK....LNISIILLPIGRAT...GHEDHVIL
Sp Type 4      ........HYNHSKKALEKF.AESISLFLEN...NPNYYVVVTSDSILP.....YEDAYY
Kp K6          .........YSKE.LLADS...............QLKRDFIVIPHM.......NEDFNLY
Kp K46         .........FSRDTLLINT...............EPKRDFIVIPHM.......NEDFSLY
Xo             .........FPADAL.................GPINKRPFAIVPHF.......NEPVEKY
Kp K14         .........YPAKQIT...................KKYTLGIIPHL........KDYAFF
Kp K64         .........YCPKK....................NKHYKVGIIPHL........KDYDYF
Kp K74         .........YCPKK....................NKHYKVGIIPHL........KDYDYF
Kp K7          ......................DEFKPLPEN....ERKGIIFIPHHHALL..........
Kp K31         ......................EEFKPLSDN....ERNGIIFIPHHHALL..........
Kp K56         ......................AEFKPLPEN....ERSGVIFIPHHHAIS..........
Kp K27         ......................PEFSPLSEK....ERKGIIFIPHHYAVH..........
Kp K36         ......................AEFKPLPED....ERDGVIFIPHHNALD..........
Rm             ......................PEFQGLRKI....YKRS..FVPHWESAI..........
Kp K8          .....KND.HMIQELSGKF...NKVVVTNNK.............................
Kp K33         ......................KRILN....................GRIKELSVKNNIL
Kp K35         ......................KRILN....................GRIKELAIKRDIL
Bf WcfO        ......................KPVLKILRNYKKNNRFTINISKVGIGRLLLITKSYLLL
```

```
                                                  200       210       220
Kp O1 WbbZ     ....NGDYWDN....................EFLARNSTRCMINFLEEYKVVNTDRLHVA
Cv             ....NGDYWDN....................EFLARNSTRCMINFLEEYKIVNTDRLHVA
Ab K20         RYINN.......................FAMNTARKRLINTGIQFINGFDDVYTTRLHGC
Ab K82         KIIDK.......................IAITYMKDKLFKQGVDFIGEYEVIYTTRLHGC
Pm O24         NNLFI.......................KKWEKHMYSFCSKGVSELNEANDITTSRLHGF
Kp K13         DIESN.........................WNKYTDAMLVRINNYFMSFDEVITSRMHGH
Kp K26         KNIIY.......................FLWDKHSSRVVNRSARYFSKYNVIITSRMHGH
Kp K30         KNISN.......................VIWYNYTYKMVQRYALYFTSHEKVVTSRMHGH
Kp K69         KDLSN.......................KIWYTYTLKMVDRYALFFTKHEKVVTSRMHGH
Sd Type 9      ..............................WDKIASDMVFKSINYFMSHNEVYSDRLHGI
Kp K12         DNYVK....................DYVLTFEKNRDRLLKEKFEEFRHAKLIITDRLHGM
Sp Pvg1p       ......LLWDP......PVAQNPDSSF....DDRGQARYEAGAEFLASARVVITDRLHAH
Kp WbmX        RETFNNTLANGIEIEF.................VTGNEILRRGIPFNSRSFVISSRYHIN
Kp K5          NDNIDWLVWDA......................RNYSIVEFMNEFVGNSEVIISSRAHGV
Sd Type 10     KKLKT.KLPDNFNLL....................FSDYVDSVNLLKNSTFHIGMRLHSI
Pa CsaB        KKLEN.EVSEVDGLCGGPSAAAEVGTV..MSLCAPLEHPQSMLQEVSQCRVLVGMRLHSL
Kp K11         LSIKK.LVQNKENYH...VVMDELNDL.............QLGTLFQQCMLTIGTRLHSA
Kp K21         LSVKN.KVHDSQCCH...VVMDEFNDY.............QLGILLSNCVFTIGTRLHSA
Ep             ITLGE.YVKQKDKYR...VIMDEFNDL.............ELGILLGECHLTIGTRLHSA
Ec K-12 WcaK   LNLRQ.HISDPARYH...VVMDELNDL.............EMGKILGACELTVGTRLHSA
Kp K32         HSLKE.QFNAHNVEC...............FIDTEVTDFYKYDQSVTDSSLVVSMRYHGV
Kp K3          RIVYNQISKNYIHLLD............RIIIKDEVLPVREYIELLKTAKIVRSGRMHSL
Kp K68         KLIYGRLVELGYSSK..............IELVESIPSTYEYLALLNNVNKVISGRMHSL
Kp K1          NLIEKELVANGLIVTK.............YYYDITKTSFEALGDIYKKTKIILSNRLHSL
Kp K58         TKIKEELLSINPTIEVE...........DYFYNISKESYMELKDIYRETDIIFSNRLHAL
Sd Type 1      TKIKEELLSINPTIEVE...........DYFYNISKESYMELKDIYRETDIIFSNRLHAL
Sd Type 2      KR....HLFADCTFT................IIIYNGNVDEFIMKWSTSKYALCTRFHSY
Ec O156        NTFST.DIRNGLKILR.................PSLSELNA..NLMLKETDYVGTRLHAG
Pm O51         SNLTN...LNNINIVP.................PRLKNYNDF..LLNNECDYIGTRLHGG
Pm O52         MSFNN.EI.NDINVIP.................ANLKSFDSLLDNHI.SLDFIGTRLHAG
Kp K70         SEVKK.YSREEIELL......DDLNVW.............EIMFIISKAKAFYGTSLHGV
Kp K80         QKVNA.CANNNFILL......DDLNVW.............EIMYIISKSKVFYGTSLHGV
Kp K72         SRIFT.LLSNASIPVA................MQNDPHVLSIMATLANAKAYIGTSLHGA
Sp Type 4      QEFRK.LVRTEDCFQ..................FKYHSPAEMTSLLKMVDVVLTCKLHVG
Kp K6          AKYKS.........................NICSPNQGAIGFTKEIVNSNFVISSSLHGV
Kp K46         KKYNN.........................NICSPKQGAISFTRQIVNSEFVISSSLHGV
Xo             SAYKE.........................HLVFPNVKPATFMSALLGVELVVSSSLHGL
Kp K14         KQ....MYGTNKNVH...............VIDLKTSDIEFVLNQMLACEKIISTSLHGV
Kp K64         KN....KYRSNKNIK...............VIDLKTKDIEFVVDEIISCEYILSTSLHGV
Kp K74         KN....KYRSNKNIK...............VIDLKTKDIEFVVDEIISCEYILSTSLHGV
Kp K7          .................SGQWELACKLSNIEFVNPQWDSKIVIQKIRHAKMVIADAMHAA
Kp K31         .................TGNWQYVCEQAGVEFVNPQWDAKIVINKIRGAKMVLADAMHAA
Kp K56         .................VGNWEEVCRLAGVEYVSPQWNAVDVINKIRNAKLVIADAMHAA
Kp K27         .................AGEWEEVCKLAGVEFVNPESDSKYVLDKIRNAKLVLADAMHAA
Kp K36         .................TGNWEEVCNRAGIEFVNPTLESKIVIQKIRHAKLVLADAMHAA
Rm             .................AGLWPAICDAVGLNYIDPRGEAKDVIRKIGQSELIVAESMHGA
Kp K8          .................PYPLSYVKSLL.AQDIFVSDTPLDYLILLANAKEVHSDRVHSC
Kp K33         RKYFDEDILSN......AVYFDQMVPY..IDADTGFKMADEYLKRLASAKLVVTSRIHTA
Kp K35         QKYFEEDVLDN......AVYIDQMVPY..ISPEVGFQKADAYLKRLSAARFVVTSRIHTA
Bf WcfO        RKLVVPDVLYNAIFI...TQFNMSNEY..SSESERFARADELLTKMASAQYVITSRIHCA
```

```
                230       240       250       260
Kp O1 WbbZ      ILASLLGKEVNFYP.NS.YYKNEAVYNYSLFNRYP.........................
Cv              ILASLLGKEVNFYP.NS.YYKNESVYNFSLLNHYP.........................
Ab K20          ILSLLLDKKIILLD.NS.YGKNKAYYDAWLKDSDN.........................
Ab K82          ILALLLNKKVILLD.NS.YGKNAGFYNAWLSECSN.........................
Pm O24          ILSYIMNKNVSVLD.NR.YGKNYSYIKKWINND...........................
Kp K13          ILCCLLEVKTKVID.NS.YGKNRGYYDSWTKYVPN.........................
Kp K26          ILSCLVNRNSKIID.NS.YGKNSGYFNEWTNKTEL.........................
Kp K30          IFSCLLSLPNDVID.NA.YGKNSGYFKEWTYDIDG.........................
Kp K69          IFSCLLSMPNELID.NA.YGKNSGYFKEWTYDISN.........................
Sd Type 9       ILAALLGKKIRCFD.NN.YKKNSRYISCWLKEYPS.........................
Kp K12          IFAYITKTPAIVFD.NS.YGKVKHSFENWLSDSKY.........................
Sp Pvg1p        ILSTLMGIPHIVVE.NSQMGKITNYHNTWLHGCTL.......DGVSVVVD..........
Kp WbmX         LIYSMLNVSGIAVYQNEYYRNKHKSVTEMGGAWSI....LNHDKLSEALD..........
Kp K5           LLPASLGVPTIAVA....IENKLLKVHDMISDGNI...IVKIAEHKAFDE..........
Sd Type 10      VFASINSVPIVPFS....YSPKINSFINTMFLDAK.....VFDGYSNIFNLSIDD.....
Pa CsaB         IYAASQEVPLAGIS....YDPKIDQFLHRLQEEAI........GTTAKLDAAR.......
Kp K11          IISMNFGTPAIAFN....YEHKSRGVMNELDMEFL......SSDINELITGS........
Kp K21          IISINFGTPAIAIN....YEHKSLGVMRGLKMDVL......SADVSDVIDGT........
Ep              IISMNFGTPAVAIN....YEHKSLGVMKQLGLPDM......ASDVQSLMDGS........
Ec K-12 WcaK    IISMNFATPAIAIN....YEHKSAGIMQQLGLPEM......AIDIRHLLDGS........
Kp K32          VLSVKNNIPVISLS....YENKMKECCRYSGILSQNID.LLNFDIERFNE..........
Kp K3           ILGHISGCKCIPYN....INKKIEHFSHEYLDHDA.........................
Kp K68          ILGHISGCRIETID....INKKIDQFMFEYSDKHP.........................
Kp K1           LFAFEYGAYPIAIG....DKDENFKVFHVLNDLKM.........................
Kp K58          LYAFESGAYPIAMG....NAEQNFKVKYVLEDTLL.........................
Sd Type 1       LYAFESGAYPIAMG....NAEQNFKVKYVLEDTLL.........................
Sd Type 2       ILARNCKQNFRVFC....YSNKIVEYIKTHSPFEM.........................
Ec O156         IRALQLRARTIIIG....VDNRAIEKAKDFNLPVL.....KRDAILELENMIVEP.....
Pm O51          IKALQNFHRTFIIG....IDNRANEKQKDFNLLVL.....KRKDINQLDDLINQN.....
Pm O52          IRALQKGKRTTIIS....VDNRAEEKKKDFNLNVI....PRNWDISNYIDFFNNS.....
Kp K70          ITAMSFGVPHYCIN.SD.IKKLTSFLNTWSISPFN.........................
Kp K80          ITAMSFCVPHYSIN.SG.IEKLTSFLQTWSIYPYN.........................
Kp K72          ITSYSFGHKVCAIS.TRKVVKLRGFLEAWMDPADY........SVSSDFDFSD.......
Sp Type 4       VVATCFNKSVIAIAC...HPEKTARYYGAIGELQR.........CESLFD..........
Kp K6           ILAEAYGIPAVFLG.AN.SGESQFKYDDYYFGTGR....NTYARAKTVEEAFDLRTDAIP
Kp K46          IIAEAYGIPAVLLE.NS.SGESAFKYDDYYLGTGR....DTYPIVHSIEDAFKVKPASSI
Xo              ILAEAYGIPAVYLD.WG.NGEDRFKYDDYYNGTGR....MQWHSGNSVEECLQLGGNGDF
Kp K14          IVGHAYDIPTIWIKHND.IDTDGFKFNDYFDSVGI....KLYKGFTNFDDIIENHADFFN
Kp K64          IVAQAYDIPTLWIKHND.INTDGIKFYDYFDSVGI....KPYDGFEDYESLINDYESAFV
Kp K74          IVAQAYDIPTLWIKHND.INTDGIKFYDYFDSVGI....KPYDGFEDYESLINDYESAFV
Kp K7           IIADAMRVPWVPVI.TS.PQINTFKWLDWTQTINLSY.EPKVLGASSFREAYRSRTLSLY
Kp K31          IIADAMRVPWLPLI.TS.PQINTFKWLDWTQTIQEKY.SPLVLGSSSLRESIRSRSLGFY
Kp K56          IIADAMRVPWVPVI.TS.PQINTFKWLDWALTINTPY.SPIDLGPSHFHESLRSNTLKYY
Kp K27          IIADAFRVPWVPMV.TS.PQINTFKWLDWTSTIEQRY.TPIVLGSSSLKEMVRSKGLFLY
Kp K36          IISDAMRVPWVPLI.TS.GQINTFKWLDWTKTVNLPY.MPIVLGSSSLKEFVRDKLLFLY
Rm              ILADAFRVPWTAVS.TS.HSINSFKWNDWAQTLGVTY.RPRRVPVSTRAEAM......IK
Kp K8           IPTLSFGGKCRIYS....HSKRVALFDN..............................V.
Kp K33          LPCLAIGTPVIFVN.GG.FKTKVDN..............CRFDGLFDFFN..........
Kp K35          LPCLAMGTPVIFVN.GG.FKTKVDN..............CRFDGLFDFFN..........
Bf WcfO         LPCLGFETPVVYIR.NL.SESKKST..............CRLGGLESLFN..........
```

```
Kp O1 WbbZ    ......KTCFIT................................................
Cv            ......KTCFIT................................................
Ab K20        ............................................................
Ab K82        ............................................................
Pm O24        ............................................................
Kp K13        ............................................................
Kp K26        ............................................................
Kp K30        ............................................................
Kp K69        ............................................................
Sd Type 9     ............................................................
Kp K12        .......IKFIEGDDLNLEN............VIKIANELVNTDKEF.............
Sp Pvg1p      ..................................SVDKALSLLLEWNEAG..........
Kp WbmX       ......HWLPLDSENYTSPNNMINKA........KEKKELFNKIIANAQN..........
Kp K5         .........ALTCFRKDKE...............QLRVNLEQEIT...............
Sd Type 10    .....LICKEINNENLNAE.........................................
Pa CsaB       ......AASHMCCLLDDVDGWREEHH........KAITLLKQEADQP.............
Kp K11        ......VVDKIDHVLENNE...............DVKNTLAQRIN...............
Kp K21        ......LINKIEDLIANMA...............DIEISLKNNLQ...............
Ep            ......IIAKVNGVLDNYE...............AVEQQVARAVE...............
Ec K-12 WcaK  ......LQAMVADTLGQLP...............ALNARLSEAVS...............
Kp K32        .........LLNSINENTN...............QVDNGFLKKMA...............
Kp K3         .................................SNISKSVYNSFGNAFSGDKG.......
Kp K68        .................................SELNANLNEKFKN..............
Kp K1         ......DHIIVNIENDDVNRYELER.........AIQSQLLNSNKWN.............
Kp K58        ......QDNFIDITVENFTGDE............AIKQIFKSRNQNL.............
Sd Type 1     ......QDNFIDITVENFTGDE............AIKQIFKSRNQNL.............
Sd Type 2     ............................................................
Ec O156       ......LITQINMPWGNIE...............KWKDQF....................
Pm O51        ......HEMKITIKNENIN...............KFKSQFI...................
Pm O52        ......YTTNIKIPLENIA...............KWRSQFE...................
Kp K70        ......ESLTINNIFNSISYIE...........SYDNTQLLESVE...............
Kp K80        ......TPITIDKISESIESSR...........CEGVNNLERATK...............
Kp K72        ......DFLRLCSESYDIN.................NDELLVSQKNMISA..........
Sp Type 4     ........SSVNSIVKKLETFHLKPI........TIPSELVLKARSS.............
Kp K6         ................DLK...............NIQAGLLKAFPFDLW...........
Kp K46        ................NVR...............HIALPLFNAFPYDLWDKL........
Xo            ................DLE...............KLQAGLLSVFPYDLW...........
Kp K14        ......SNKDIYLPTVDLK...............NMQKRLLEVAPFD.............
Kp K64        ......KHANISKITTDLK...............KMQDNLLSVAPFP.............
Kp K74        ......KHANISKITTDLK...............KMQDNLLSVAPFP.............
Kp K7         GEKYYIDTLSVDDAVEDFYRKRNLKSKKWWRSYKTTTEKMVFSGPNKLLSNLGFD..KHF
Kp K31        GEDYYIKNLTIDSALSDFYKKRAYKSKIWWPKYNKAAKKLVSTIPDKIALSLGETTLNKF
Kp K56        GEKYKLSDISPESAIKDFYFKRKYKSSKLWPTYSRIARRLTYNLPYKILSSLKDSEIKNK
Kp K27        GEKYYNNNCDVESSIKQFKIQRKIKSHTLWPLYRKPASFLANRVAINAAS.LVEKIDRSL
Kp K36        GEKYFVPSLTEKAALNKFKIHRLMKSNFLWRYYFKAAKILFYKIPMILVSMLGARFVSRK
Rm            GARFWGMDFQAKEPQPEDPNRR............QIDGDLAVAEREPRQTSLRAAA....
Kp K8         ......NLSNVKNEVVGIT...............NLSIYQDEQIN...............
Kp K33        .........RIDIDSAGNSSTNFSFVGNKI....TRMTELKNKDLH..............
Kp K35        .........RIDVEASGKSHCNFNFEGNKI....GFNTILTNKDLH..............
Bf WcfO       .........VITVKGENVTS...NFFDGLF....KRDSSFKNKIDF..............
```

```
Kp O1 WbbZ    ...............................................AS
Cv            ...............................................TS
Ab K20        ...............................IKVVE...........SC
Ab K82        ...............................IVEVKS..........VN
Pm O24        .................................................
Kp K13        ...............................CELI............TN
Kp K26        ...............................SSLL.........LKHEK
Kp K30        ...............................TKLL............EE
Kp K69        ...............................AKLI............EE
Sd Type 9     ...............................LKNAS...........DF
Kp K12        ...............................VFNVEDVFEPLINIT.DK
Sp Pvg1p      ...............................................YF
Kp WbmX       .......................KDKKSI....SLESALA.VINKF.IT
Kp K5         .......................KNHQKVKD..AIDEFTKWLKD...NV
Sd Type 10    .......................KDRALI....TIKHVL........SF
Pa CsaB       ...............................AQQIVQWLR.....H.KA
Kp K11        .......................ATRVRGYN..NIKEILEGLK......
Kp K21        .......................DAKIIGRT..MIENILSEIDR.....
Ep            .......................QERNLGNK..ITADVLNSLG......
Ec K-12 WcaK   .......................RERQTGMQ..MVQSVLERIGE...VK
Kp K32        ...................................SSVVD.FITVY.SR
Kp K3         ...............................................SK
Kp K68        ...............................................II
Kp K1         ......................GND.......AMCDFI...........
Kp K58        ......................TKD..INYNMIKISDFIM.......VE
Sd Type 1     ......................TKD..INYNMIKISDFIM.......VE
Sd Type 2     ....................................EYVD.......VA
Ec O156       .................................................
Pm O51        ................................................I
Pm O52        .................................................
Kp K70        .......................NTQGLIMS..SLNDIVNM......LE
Kp K80        .......................NAQSIIRA..SLDDIAA.......IL
Kp K72        .................................ELSQYL..........
Sp Type 4     ...............................LDYLDLFLEGLVR...ES
Kp K6         .................................................
Kp K46        ...............................................KK
Xo            .................................................
Kp K14        .................................VLSNYYN.......LF
Kp K64        .................................VLDKFK..........
Kp K74        .................................VLDKFK..........
Kp K7         DEPYQANVVKNLMGLKESSGFLSEDKYFYRNIERLLECKESLLK...LK
Kp K31        DLKYIESAVKMVESCKTRQPFLSNDNVFYENVSKLMSCIDKVKK.Y.KI
Kp K56        NLVYTESAAERLIKVKNSSGFLSDDVVFFNNVDGLLSKLDILKQ...HK
Kp K27        NQKFIDESVKIMISASQQHGFLSDDKIFESNLGRLYDCLYLLKK.....
Kp K36        DENFINLATEKLSEAIVSKAYLSQDSIFFDNQKKLEIALSNVVKKY.TE
Rm            KRALAAPATLALWQASRAAPQLSKDSALAERKERFRTVLDGIRRD..YF
Kp K8         .................................NLRNILR.......EI
Kp K33        ...............................VPFANELIEKCKEFVIKN
Kp K35        ...............................VPYANDLINKCLSFV.KS
Bf WcfO       ...............................VEYRDRLINICNKFM.MS
```

Figure S8. Multiple sequence alignment of WbbZ orthologs. Multiple sequence alignment of candidate pyruvyltransferases was performed with in MAFFT (6) and visualized using Espript (7). Conserved $N^{29}xG^{31}D^{32}$, R^{121}xxx(S/T), and H^{226} were shown to be present in a similar location within the predicted structure of select orthologs and the crystal structures of WbbZ and Pvg1p (**Figure 6b**). Accessions and species/strain information are listed in Table S2.

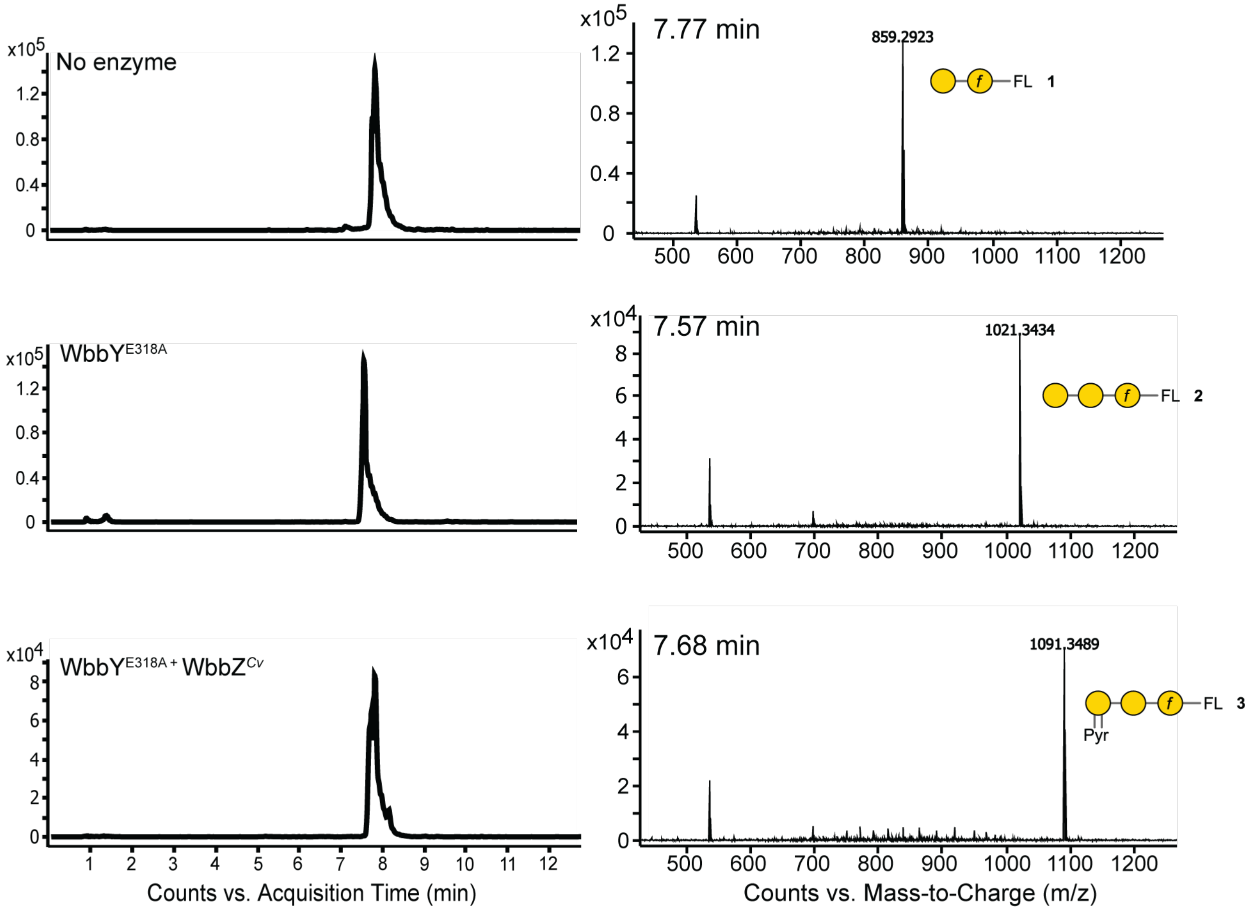

Figure S9. Mass spectrometry confirmation of the structure of the *in vitro* product synthesized by the *Caudovirales* WbbZ enzyme. The acceptor and reaction products with visualized by PAGE (Figure 5) and then subjected to analysis by MS. Extracted ion chromatograms (EICs) are shown in the left panel and the ESI mass-spectra on the right. Triplicate reactions were examined by PAGE with the same results and products from one experiment were analyzed by MS.

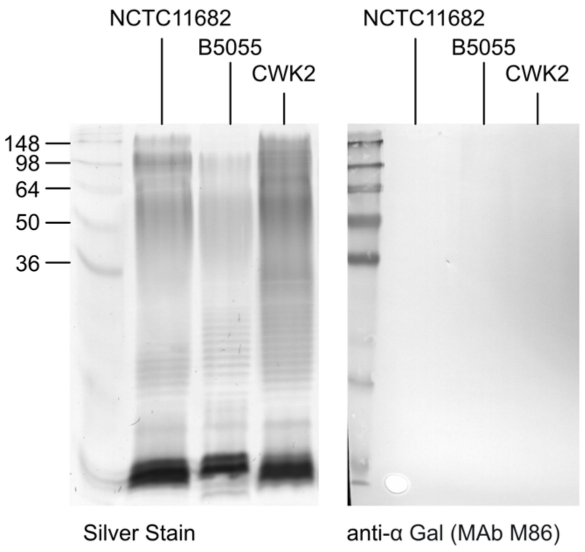

Figure S10. ***K. pneumoniae*** **O1 LPS is not recognized by validated MAbs specific for the α-Gal-1,3-Gal epitope.** Whole cell lysates were probed in western immunoblots using M86 (Enzo Life Sciences). The reactions were performed in triplicate with the same outcome.

Table S1. ^{1}H and ^{13}C NMR data (δ, ppm) for purified OPS, compound 2 and the product of the *in vitro* WbbZ reaction 3.

Residue		*H-1*<br>C-1	*H-2*<br>C-2	*H-3*<br>C-3	*H-4*<br>C-4	*H-5*<br>C-5	*H-6a*<br>C-6	*H-7 (6b)*<br>C-7	*H-8a,b*<br>C-8
***K. pneumoniae* OPS**									
3,4-(*S*)-Pyr (major)				*1.61*					
		178.6	109.1	24.6					
4,6-(*R*)-Pyr (minor)[a]				*1.48*					
		176.9	102.1	26.4					
β-Gal*p*3,4(*S*-Pyr)-(1→	**T**	*4.69*	*3.56*	*4.22*	*4.13*	*4.04*	*3.85*	*3.90*	
		104.4	74.7	79.7	76.0	74.2			
β-Gal*p*-(1→	**D′**	*4.64*	*3.63*	*3.67*	*3.94*				
		105.5	72.4	73.9	69.9				
→3)-β-Gal*p*-(1→	**D**	*4.70*	*3.77*	*3.80*	*4.20*	*3.70*	*3.80*		
		105.4	70.9	78.5	66.1	76.0	62.2		
→3)-α-Gal*p*-(1→	**C**	*5.19*	*4.07*	*4.15*	*4.29*	*4.25*	*3.75*		
		96.5	68.5	80.3	70.3	71.8	62.2		
→3)-β-Gal*f*-(1→	**B**	*5.24*	*4.42*	*4.09*	*4.28*	*3.89*	*3.69*	*3.72*	
		110.5	80.9	85.7	83.2	72.0	64.0		
→3)-α-Gal*p*-(1→	**A**	*5.10*	*3.97*	*3.94*	*4.16*	*4.14*	*3.77*		
		100.6	68.4	78.2	70.4	72.4	62.4		
→3)-α-Gal*p*-(1→	**A″**	5.47	3.93	3.85	4.14				
		100.1	68.7	78.2	70.2	71.9			
→3)-β-Glc*p*NAc-(1→	**F**	*4.67*	*3.85*	*3.80*	*3.73*	*3.53*	*3.95*		
		101.7	55.3	80.5	72.1	76.7	61.9		
→3)-β-Glc*p*NAc-(1→	**F′**	*4.61*	*3.85*	*3.78*	*3.72*	*3.51*	*3.95*		
		103.0	55.4	80.5	72.1	76.7	61.9		
→5)-Kdo[b]	**L**			*3.13*	*4.38*	*4.13*	*4.19*	*4.00*	*3.64, 3.67*
				43.6	78.7	89.5	77.3	85.1	
α-Hep-(1→	**P**	*5.18*	*3.93*	*3.76*	*3.85*	*3.59*			
		95.4			67.6	73.1			
**compound 2**[c]									
β-Gal*p*-(1→	**T**	*4.55/*<br>*4.59*	*3.60/*<br>*3.62*	*3.63/*<br>*3.65*	*3.91/*<br>*3.93*	*3.64/*<br>*3.67*	*3.74/*<br>*3.76*	*3.74/*<br>*3.76*	
		105.6	72.2	73.7	69.7	76.2	62.1		
→3)-α-Gal*p*-(1→	**A**	*5.06/*<br>*5.09*	*3.98/*<br>*4.01*	*3.98/*<br>*4.01*	*4.25/*<br>*4.27*	*4.05/*<br>*4.09*	*3.74/*<br>*3.76*	*3.74/*<br>*3.76*	
		100.8	68.5	80.4/<br>80.3	70.3	72.3	62.1		
→3)-β-Gal*f*-(1→	**B**	*4.96/*<br>*5.03*	*4.21/*<br>*4.26*	*4.04/*<br>*4.08*	*4.14/*<br>*4.17*	*3.85/*<br>*3.87*	*3.66/*<br>*3.70*	*3.66/*<br>*3.70*	
		108.8/	80.6	85.9/	83.1	72.1	64.0		

		108.7		85.7				
**compound 3**								
3,4-(*S*)-Pyr				*1.59*				
		178.7	109.0	24.7				
β-Gal*p*3,4(*S*-Pyr)-(1→	**T**	4.53	3.51	4.13	4.06	3.90	3.81	3.84
		104.5	74.7	79.7	76.0	74.1	62.0	
→3)-α-Gal*p*-(1→	**A**	5.05	3.98	3.95	4.18	4.00	3.71	3.74
		100.9	68.6	80.4	70.3	72.4	62.4	
→3)-β-Gal*f*-(1→	**B**	4.93	4.20	4.04	4.11	3.86	3.67	3.67
		108.8	80.9	86.1	82.6	72.0	64.3	

[a] The location of minor Pyr is unknown. Indicated position of attachment and absolute configuration are based on Pyr chemical shifts only.
[b] Residue **L** was tentatively identified as one of stereoisomers of 4,7-anhydro-Kdo. The signals for a different Kdo form (residue **L′**) were present in the spectra at δ_H/δ_C 4.20/87.1, 4.30/78.4, 4.51/77.3 and δ_H 4.41, and but were not assigned. The linkages **F**→**L** and **F′**→**L′** were confirmed by HMBC data.
[c] Two series of signals were present in the spectra, both series corresponding to the same trisaccharide moiety structure. We attribute the signal splitting to a potential aggregation of **2** in D_2O as it has limited solubility. The split signals merged upon conversion of **2** to **3**.

Table S2. Identification of *wbbY* and *wbbZ* genes in other bacterial species

Strain/ Genome accession	WbbY/ WbbZ accession	Gene organization	WbbY/WbbZ identity shared with NCTC11682	O2a cluster
Escherichia coli F8188-41 (O19ab)/ KJ451391	AHZ58497.1/ AHZ58498.1	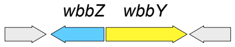	96%/94%	Unknown
Escherichia coli RHB33-C12 (O51) plasmid pRHB33-C12_3/ CP057196	QME46802.1/ QME46803.1	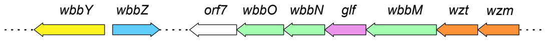	99.6%/99%	*rfb*[2a] genes present on plasmid in addition to *gmlABD*
Escherichia coli GD33 (O79)/ CP076646	QWU38941.1/ QWU38942.1	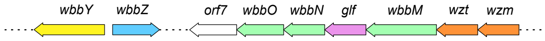	99.6%/99%	Yes
Escherichia coli EC21 CP060899	UND24401.1/ UND24402.1	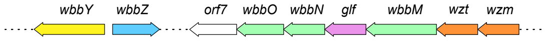	99.6%/99%	Yes
Escherichia coli C21 (O51)/ CP052877	QJP81697.1/ QJP81696.1	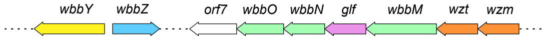	99.6%/99%	Yes
Escherichia coli WCHEC025985 (disrupted O165)/ CP043284	UNC35906.1/ UNC35907.1	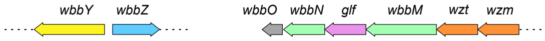	99.5%/99%	Disrupted
Escherichia coli RHB24-C01 (O8)/ CP057510	QMJ17736.1/ QMJ17737.1	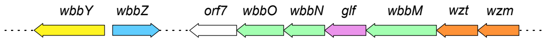	99.6%/99%	Yes
Escherichia coli YD786/ CP013112	WbbY disrupted/ ALN45138.1	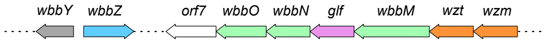	-/99%	Yes
Escherichia coli THO-010 (O130)/ AP022540	BBW71254.1/ BBW71255.1	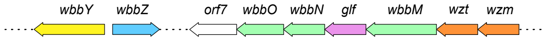	99.6%/99%	Yes
Escherichia coli CFSAN061769 (O5)/ CP042969	QEH84816.1/ QEH84817.1	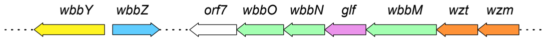	99.6%/99%	Yes
Escherichia coli MRSN346647 (O153)/ CP018206	API46670.1/ API46669.1	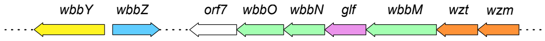	99.6%/99%	Yes
Escherichia coli SMS-3-5 (O153)/ CP000970	ACB18668.1/ ACB16956.1	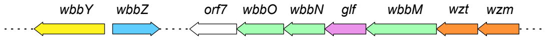	99.6%/99%	Yes
Escherichia coli XDL (O153)/ CP043033	QFI49926.1/ QFI49925.1	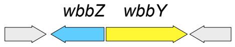	99.6%/99%	No
Escherichia coli H17 (O153)/ CP021193	AWR83820.1/ AWR83819.1	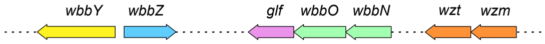	99.6%/99%	Disrupted

Raoultella terrigena<br>NCTC 9997/<br>LR134253.1	A0A3S4KBB7/<br>VED46416.1	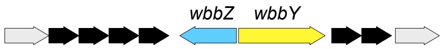	96%/93%	Disrupted
Citrobacter freundii<br>RHBSTW-00488<br>plasmid pRHBSTW-<br>00488_5/<br>CP055470.1	QLO45501.1/<br>QLO45502.1	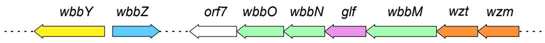	96%/93%	Yes
Serratia marcescens<br>UMH6/<br>CP018926.1	ASM11289.1/<br>ASM14987.1	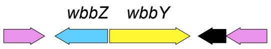	63%/64%	Yes
Pseudochrobactrum<br>*algeriensis*<br>C150915_17 plasmid<br>pB17_3/<br>CP075350.1	QVQ38603.1/<br>QVQ38602.1	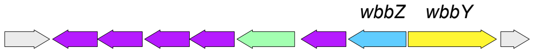	21%/39%	No
Lysobacter<br>*enzymogenes* C3/<br>CP013140.1	ALN59219.1/<br>ALN59218.1	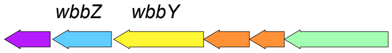	33%/39%	No
Acetobacter persici<br>TMW2.1084/<br>CP014687.1	AQT05624.1/<br>AQT05623.1	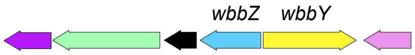	24%/31%	No

Insertion sequence/transposon

Hypothetical

Housekeeping

Glycosyltransferase

Nucleotide sugar biosynthesis

ABC transporter

- - - Several transposable elements

Table S3. Pyruvate-containing polysaccharides from bacteria with available genome sequences.

Source	Structure	Genome accession/ PVTase accession	Gene name
K. pneumoniae O1	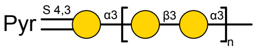	UGMM01000004.1/ STV47229.1	*wbbZ*
Caudovirales sp.	Unknown	BK049963.1/ DAJ90914.1	
K. pneumoniae O2ac	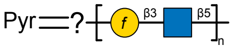	MG602074.1/ AVA30549.1	*wbmX*
A. baumanii K20	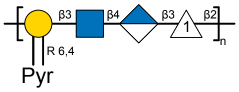	JQ684178.2/ AIT56457.1	*ptr2*
A. baumanii K82	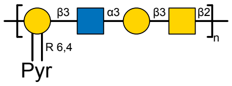	KC526908.1/ AHB32560.1	*ptr5*
E. coli O156	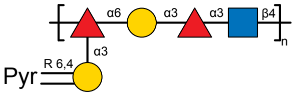	CAADJH010000002.1/ VFS84308.1	*csaB*
P. mirabilis O24	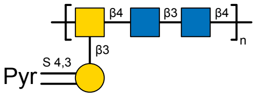	KY710701.1/ AXY99566.1	*orf03*
P. mirabilis O51	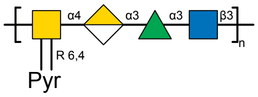	KY710719.1/ AXY99831.1	*orf09*
P. vulgaris O52	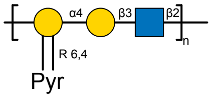	KY710720.1/ AXY99845.1	*orf05*
K. pneumoniae K1	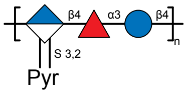	CP016814.1/ AOE32221.1	
K. pneumoniae K3	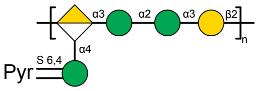	LT174553.1/ CZQ24409.1	*wclV*
K. pneumoniae K5	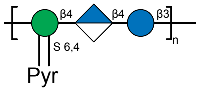	AB371292/ BAI43762.1	*kp5c*
K. pneumoniae K6	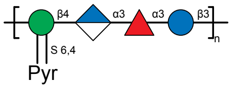	LT174571.1/ CZQ24758.1	*wclT*

K. pneumoniae K7	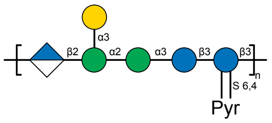	AB924550.1/ BAT23235.1	*wcuJ*
K. pneumoniae K8	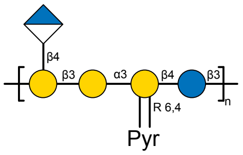	AB924551.1/ BAT23251.1	*wcsI*
K. pneumoniae K11	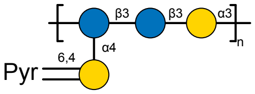	LT174533.1/ CZQ24036.1	*wcaK*
K. pneumoniae K12	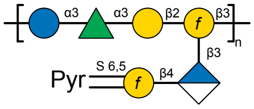	AB924554.1/ BAT23304.1	*wckH*
K. pneumoniae K13	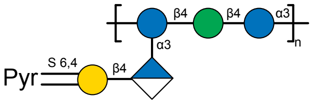	AB924555.1/ BAT23323.1	*wcuL*
K. pneumoniae K14	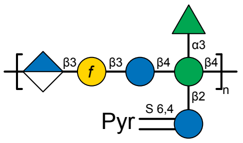	AB371294.1/ BAI43800.1	*kp14c*
K. pneumoniae K21	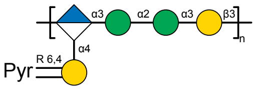	AB924560.1/ BAT23419.1	*wcuA*
K. pneumoniae K26	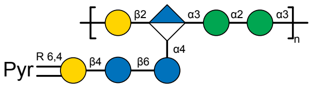	AB924564.1/ BAT23491.1	*wcuL*
K. pneumoniae K27	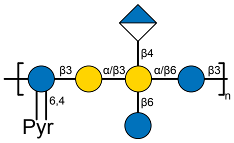	LT174546.1/ CZQ24281.1	*wcuJ*
K. pneumoniae K30	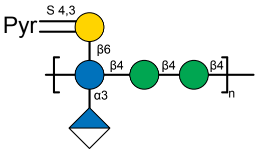	AB924568.1/ BAT23553.1	*wcuL*
K. pneumoniae K31	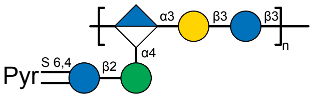	LT174550.1/ CZQ24358.1	*wcuJ*
K. pneumoniae K32	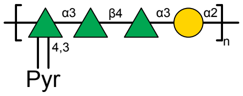	LT603726.1/ SCA96194.1	*wctJ*

K. pneumoniae K33	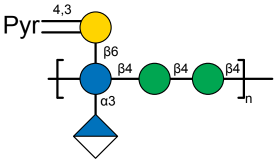	AB924571.1/ BAT23618.1	*wckK*
K. pneumoniae K35	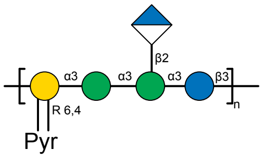	AB924573.1/ BAT23658.1	*wckK*
K. pneumoniae K36	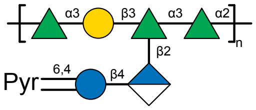	AB924574.1/ BAT23682.1	*wcuJ*
K. pneumoniae K46	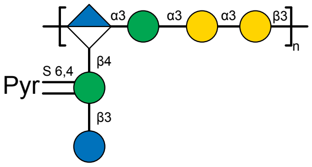	LT174557.1/ CZQ24490.1	*wclT*
K. pneumoniae K56	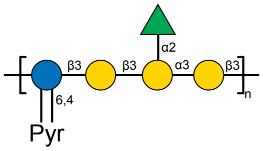	AB924593.1/ BAT24054.1	*wcuJ*
K. pneumoniae K58	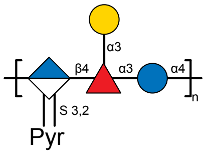	LT174567.1/ CZQ24668.1	*wclY*
K. pneumoniae K64	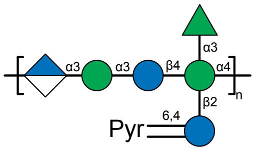	AB924600.1/ BAT24181.1	*wcoV*
K. pneumoniae K68	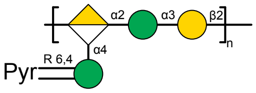	AB924604.1/ BAT24268.1	*wclV*
K. pneumoniae K69	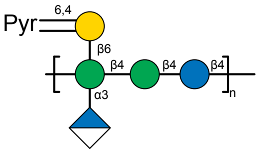	AB924605.1/ BAT24284.1	*wcuL*
K. pneumoniae K70	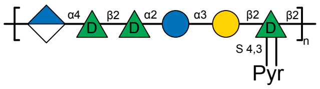	AB924606.1/ BAT24312.1	*wcuN*

K. pneumoniae K72	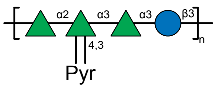	AB924608.1/<br>BAT24351.1	*wcqL*
K. pneumoniae K74	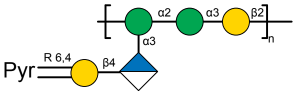	AB924600.1/<br>BAT24181.1	*wcoV*
K. pneumoniae K80	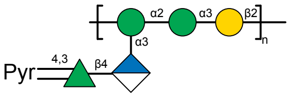	AB924611.1/<br>BAT24411.1	*wcuN*
S. pombe	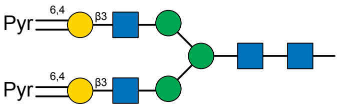	CU329670.1/<br>CAB52171.1	*pvg1p*
S. dysenteriae Type 1 = *E. coli* O149	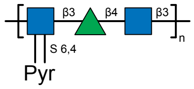	DQ868764.1/<br>ABI98965.1	*wbuT*
S. dysenteriae Type 2 = *E. coli* O112ac	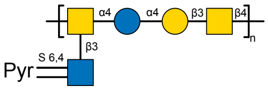	EU296404.1/<br>ACD37009.1	*wfeN*
S. dysenteriae Type 9	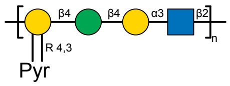	EU296416.1/<br>ACD37114.1	*wffR*
S. dysenteriae Type 10	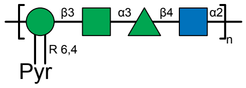	EU294178.1/<br>ACA24911.1	*wffS*
E. pyrifoliae Ep1/96	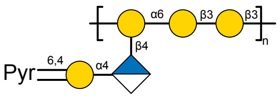	NC_012214.1/<br>WP_012667715.1	*wcaK*
S. pneumoniae Type 4	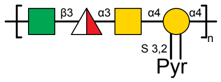	AF316639.1/<br>AAK20675.1	*wciM*
P. alvei SCWP	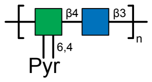	LS992241.1/<br>SYX86526.1	*csaB*
B. fragilis PSA	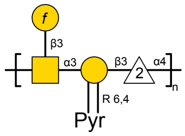	AF189282.1/<br>AAK68918.1	*wcfO*
X. oryzae pv. Oryzae Xanthan gum	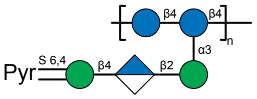	AE013598.1/<br>AAW76423.1	*gumL*
E. coli K-12 Colanic acid	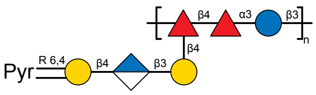	CP047127.1/<br>QHB68711.1	*wcaK*

R. meliloti Succinoglycan	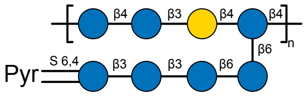	Z22646.1/<br>CAA80360.1	*exoV*

Table S4. Primer sequences. The primers used for Gibson assembly contain regions with homology to genes marked in lower case and regions with homology to plasmid sequences in upper case. Ribosome binding sites are indicated in bold and sequences encoding His$_6$-tags are in italics. For mutagenesis primers, the mutations are in indicated in lower case.

Plasmid generated	Purpose	Primer sequence
pWQ1111	Forward primer for Gibson assembly of His6-WbbY in pBAD18—Kan	CATACCCGTTTTTTTGGGCTAGC**aggaggaattcacc**atg*catcaccatcaccatcac*accaatatgaagttaaaatttgatttgcttctaaaatc
pWQ1111	Reverse primer for Gibson assembly of His6-WbbY in pBAD18—Kan	TGCCTGCAGGTCGACTCTAGAtcaacttgccgtaataaagcatgtttttg
pWQ1112	Forward primer to create stop codon at position 723 of WbbY	AGGCGCAAAAtaAGGCACCAGC
pWQ1112	Reverse primer to create stop codon at position 723 of WbbY	TGGACATGAATTGCTTCATAC
pWQ1113	Forward primer to clone His6-WbbZ in pBAD18-Kan	gatcGCTAGC**aggaggaattcacc**atg*catcaccatcaccatcac*accaatatgaagttaaaatttgatttgcttctaaaatc
pWQ1113	Reverse primer to clone His6-WbbZ in pBAD18-Kan	gatcTCTAGAtcaacttgccgtaataaagcatgtttttg
pWQ1114	Forward primer for E318A mutation of WbbY in pWQ1112	TCACTTGTAGcAAACTCACCC
pWQ1114	Reverse primer for E318A mutation of WbbY in pWQ1112	TGGAATGATGACTAATACATTTTTTC
pWQ1127	Forward primer for Gibson assembly of His6-WbbZ^{Cv} in pBAD18-Kan	CATACCCGTTTTTTTGGGCTAGC**aggaggaattcacc**ATG*catcaccatcaccatcac*gccaatgcagagttaaaatttgatttatttc
pWQ1127	Reverse primer for Gibson assembly of His6-WbbZ^{Cv} in pBAD18-Kan	TGCCTGCAGGTCGACTCTAGAtcaacttgtcgtaataaagcatgtttttg

Methods

Bacterial strains and growth conditions

The *K. pneumoniae* O1 strains used were CWK2 (O1:K$^-$, derived from 889/50, O1:K20), B5055 (O1:K2 reference strain), and NCTC11682 (O1:K2) (8), and NCTC11682 (O1:K$^-$) Δ*cps*, which the the *cps* cluster was replaced with a kanamycin resistance cassette via λ-Red homologous recombination (9). *E. coli* DH5α (*deoR recA1 endA1 hsdR17* (r_K^-, m_K^+) *gal*$^-$ *phoA supE44 thi*$^-$ *gyrA96 relA1*) (10) was used for cloning and expression of *Caudovirales wbbYZ,* and *E. coli* TOP10 (F$^-$, *mcrA*, Δ(*mrr-hsdRMS-mcrBC*), φ80, *lacZ*Δ*M15*, Δ*lacX74*, *deoR*, *nupG*, *recA1*, *araD139*, Δ(*ara-leu*)7697, *galU*, *galK*, *repsL*(Str^R), *endA1*) (Invitrogen) was used for protein production.

Production of O1-specific monoclonal antibodies

Monoclonal antibodies were generated against an O1 polysaccharide: protein conjugate antigen produced by *in vivo* N-glycosylation using the *Campylobacter jejuni* PglB oligosaccharyltransferase and AcrA as the protein carrier as described previously (11). O1 antigen synthesis in the glycoengineered strain was achieved using the O2a biosynthesis locus (SI Figure S3) from *K. pneumoniae* NUHL24835 (GenBank CP014004) and the *wbbY-wbbZ* O1 locus from *K. pneumoniae* AKPRH07048 (GenBank LT174607). The bioconjugate was purified by means of an immobilized metal affinity chromatography followed by an anion exchange chromatography. The O1-AcrA bioconjugate was used to immunize rats and isolate monoclonal antibodies according to the established hybridoma techniques using the service at GenScript. Briefly, two Wistar Rats were each given a primary immunization with 10 μg (13.7 μL) conjugate, followed by two boosts with 5 μg conjugate at days 14 and 28. The rat with the highest O1-specific antibody titre in ELISA was selected for the production of hybridoma cells. Hybridoma clones were screened by analysing O1-specific reactivity via ELISA and two positive clones were selected for two further subcloning rounds; mAbs563 and 576 were purified from clones 11E10 and 1B3, respectively. Their specificities were examined by western immunoblotting.

SDS-PAGE and western immunoblotting

A standard SDS-PAGE method exploiting SDS/proteinase K-treated whole-cell lysates (12) was used to examine cell surface polysaccharides. Cell suspensions equalized by $OD_{600,}$ resuspended in SDS-PAGE sample buffer and boiled for 10 min. Samples were treated with proteinase K, separated by SDS-PAGE and subsequently stained with silver or transferred to a nitrocellulose membrane for 45 min at 200 mA. Immunoblots were blocked in 5% skim milk (w/v) and probed with primary antibody using dilutions as follows: 1:1000 Rabbit anti-O2a (8), 1:250 mAb576 (2.8μg/mL), 1:500 mAb563 (1.0 μg/mL). A validated monoclonal antibody recognizing the α-Gal-1,3-Gal epitope was obtained from Enzo Life Sciences (https://www.enzolifesciences.com/ALX-801-090/alpha-gal-epitope-galalpha1-3galbeta1-4glcnac-r-monoclonal-antibody-m86/) and used at a dilution of 1:100. Goat anti-rabbit alkaline phosphatase (Cedarlane) or goat anti-mouse alkaline phosphatase (Jackson Laboratories) secondary antibodies were used at 1:3000 dilutions, and 5-bromo-4-chloro-3-indolyl phosphate/ nitro-blue tetrazolium (BCIP/NBT; Sigma) was employed for detection.

Isolation of OPS

To eliminate potentially contaminating K2 capsular polysaccharide, OPS was prepared from NCTC11682Δ*cps*. NCTC11682Δ*cps* was grown in a bioreactor vessel with acid-base feed for pH control and 1 L/min air flow rate for 24 h at 28°C. The culture medium (1.3 L) contained 24 g/L yeast extract, 12 g/L soy peptone, 50 g/L glycerol, 10 mM $MgCl_2$, in phosphate buffer pH 7, supplemented with 50 µg/mL kanamycin and antifoam 204. Cells were harvested by centrifugation and the LPS was extracted using hot aqueous phenol as described elsewhere (13), filtered through a 0.45-µm syringe filter, and purified by size exclusion chromatography on a Sepharose CL-6B column, eluted in 0.2 M NaCl. UV absorbance was monitored at 206, 230, and 280 nm and fractions were analyzed by SDS-PAGE and anti-O1 immunoblotting (14). The LPS-containing fractions were pooled, resuspended in 3 M sodium acetate, precipitated via cold ethanol, resuspended in H_2O, and finally freeze-dried. 80 mg of LPS was suspended in 2 mL of 2% v/v acetic acid and hydrolyzed at 100 °C until a precipitate formed (4.5 h). After removal of the precipitate by centrifugation, and washing the pellet in 2% acetic acid, the pooled supernatant was subjected to size exclusion chromatography on a Sephadex G-50 superfine column eluted with 50 mM pyridinium acetate buffer, pH 4.5, at flow rate 0.6 ml/min, with detection by Smartline 2300 refractive index detector (Knauer). Fractions corresponding to the early (major) peak were pooled, concentrated, and lyophilized. The yield of OPS was 21 mg.

NMR spectroscopy

Prior to analysis, OPS and compounds **2** and **3** were deuterium-exchanged by lyophilizing twice from 99.9% D_2O. ^1H and ^{13}C NMR spectra were recorded at 50 °C (OPS) or 30 °C (**2** and **3**) in D_2O, using a Bruker Avance III 600 MHz spectrometer equipped with a 5 mm TCI cryoprobe. Sodium 3-trimethylsilylpropanoate-2,2,3,3-d_4 was used as an internal chemical shift reference (δ_H = 0 ppm, δ_C = –1.6 ppm). Two-dimensional NMR spectra were obtained using standard Bruker software, and the Bruker TopSpin program was used to acquire and process the NMR data. ^1H and ^{13}C NMR chemical shifts were assigned using 1D selective TOCSY and ROESY experiments, 2D ^1H,^1H COSY, TOCSY, NOESY, ^1H,^{13}C HSQC, HMBC and HSQC-TOCSY experiments. A mixing time of 100 ms (OPS) and 80 ms (**2** and **3**) was used in TOCSY experiments, 150 ms (OPS) in NOESY and 200 ms (**2** and **3**) in ROESY experiments. The Bruker TopSpin program was used to acquire and process the NMR data.

Gene cloning and expression of recombinant proteins

Plasmid pWQ288 (which carries the *rfb*^{2a} locus conferring O2a OPS production has been described elsewhere (15). DNA fragments obtained by PCR using KOD polymerase with primers listed in Table S4 and cloned in pBAD18-Kan (16). Plasmid construction was performed using restriction enzyme digest followed by ligation with T4 DNA ligase, or by Gibson assembly (New England Biolabs). The *wbbY* gene was amplified from NCTC11862 template DNA and cloned in plasmid pWQ1111. Previous work showed the localization of CWK2 WbbY to the membrane (3), so a soluble truncation of His_6-WbbY (containing residues 1-722) was generated by amplifying the truncated fragment for pWQ1111, creating pWQ1112. The DNA fragment encoding His_6-WbbZ amplified using NCTC11682 template and cloned in plasmid pWQ1113. Inverse PCR was performed on pWQ1112 with primers containing the desired mutations to encode an

E318A variant resulting on pWQ1114. WbbYZ orthologs were identified in *Caudovirales sp.* and the genes encoding WbbY (DAJ90913.1) and $WbbZ^{Cv}$ (DAJ90914.1) were synthesized by GeneArt on a single DNA fragment. pWQ1115 was derived by cloning the fragment in pBR322 (17). The *wbbZ*^{Cv} *orf* was subcloned into pBAD18-Kan to create plasmid pWQ1127, expressing His_6-$WbbZ^{Cv}$.

For protein production *E. coli* TOP10 transformants containing pWQ1112 or pWQ1115 were grown to OD_{600} ~0.6 and 0.2% L-arabinose was added to induce expression during growth overnight at 18 °C. Cells were collected and resuspended in buffer A (50 mM Tris pH 7.5, 500 mM NaCl) containing 10 mM Imidazole and lysed using an EmulsiFlex-C3 (Avestin). Cell-free and membrane-free lysates were obtained by spins at 12,000 × *g* and 100,000 × *g.* Lysates were passed through Ni-NTA agarose (Qiagen) and washed with buffer A containing 10 mM imidazole, followed by a wash in buffer A with 50 mM imidazole and elution in buffer A containing 250 mM imidazole. Elutions were combined and concentrated using a Vivaspin 10,000 MWCO concentrator (Sartorius). Concentrated protein was buffer exchanged into buffer B (50 mM Tris pH 7.5, 150 mM NaCl) using a PD10 column (Cytiva). Protein concentration was determined using a Nanodrop (ThermoFisher) with theoretical extinction coefficients produced by ProtParam.

***In vitro* enzyme reactions**

In vitro reactions with purified proteins were performed in 100 mM Na HEPES buffer pH 7.5 with 10 mM $MgCl_2$. Standard reactions contained 0.1 mM acceptor, 10 mM UDP-Gal, 10 mM PEP and 5 μM WbbY and 2.5 μM WbbZ and 2.5 μM $WbbY^{E381A}$. Reactions were performed for 15 minutes at 30 °C and stopped by addition of an equal volume of loading buffer before analysis by SDS-PAGE. The *Caudovirales* $WbbZ^{Cv}$ enzyme was examined using the same approach except the source of enzyme was a cell free lysate from *E. coli* DH5α transformed with plasmids pWQ1127 ($WbbZ^{Cv}$) or pWQ1114 ($WbbY^{E318A}$). The reactions included 25 μM compound **1**, 1mM UDP-Gal*p,* 10mM PEP and 10ug of total protein for 1hr incubation. A reduced amount of compound **1** was used in reactions using lysates to promote complete conversion to product. In addition, a lower amount of UDP-Gal donor was used to prevent small amounts of multiple Gal*p* transfers possible with the $WbbY^{E318A}$ variant in *in vitro* reactions with high donor concentrations.

Mass spectrometry was used to determine the identity of the reaction products. Scaled up reactions to produce **2** contained 5 μM $WbbY^{E318A}$, 1 mM UDP-Gal and 1 mM acceptor. To generate **3**, reactions contained 1 mM **2**, 10 mM PEP and 5 μM WbbZ. All scaled up reactions were performed in total volume of 1.7 ml at 30 °C for 18 h to ensure full conversion. Protein was removed by precipitation with cold acetonitrile and samples were concentrated by vacuum centrifugation. **2** was bound to SepPak C18 Plus cartridge (Waters) equilibrated with water. The cartridge was washed with water and **2** was eluted in 50% v/v acetonitrile. **3** was purified by size exclusion chromatography using a Superdex 200 Increase 10/300 GL column with 100 mM ammonium acetate as the elution buffer. Elution was monitored by UV absorbance at 491 nm. Peak fractions were pooled and analyzed by SDS-PAGE. The major fraction containing **3** was desalted using SepPak C18 Plus cartridge as described above. Following SepPak purification **2** and **3** were concentrated by vacuum centrifugation and lyophilized.

Mass spectrometry

LC-MS was performed at the Advanced Analysis Centre (University of Guelph) on an Agilent 1260 HPLC interfaced with and Agilent UHD 6530 Q-TOF mass spectrometer. Purified compounds **2** and **3** were dissolved to 0.1mM in 50% ACN and 10µL of sample was injected and separated on a C18 column (Agilent Poroshell 120, 50 × 4.6 mm^2 2.7 µm) with the following solvents: 0.1% formic acid (A) and acetonitrile with 0.1% formic acid (B). The products were eluted in a gradient using 5% B increasing to 100% in 15 min. Flow rate was maintained at 0.4mL/min. The electrospray capillary voltage was set to 4.0kV with a drying gas temperature of 250°C at a flow rate of 8 L/min. The nebulizer pressure was 30 psi and the fragmentor was set to 160. Nitrogen was used for nebulizing, drying and collision-induced dissociation. The mass-to-charge ratio was scanned across the m/z range of 500–2,250 m/z in 4 GHz (extended dynamic range positive-ion auto tandem mass spectrometry mode). The instrument was externally calibrated with the electrospray ionization TuneMix (Agilent). Mass spectrometry data analysis was carried out using Agilent MassHunter Qualitative Analysis v.10.0.

Bioinformatics and structural models

The *wbbY* and *wbbZ* genes from KJ451390 (https://www.ncbi.nlm.nih.gov/nuccore/KJ451390.1) were employed as query template. The *Klebsiella* assemblies from a reported diverse collection (18) were searched for *wbbY* and *wbbZ* matches using blastn with standard setting. The threshold for an aligned match was set at covering a minimum sequence length of 80% of the interrogated genes. *In silico* serotyping was confirmed using the current version of Kaptive (https://github.com/katholt/Kaptive/wiki), v2.0. The results are provided as a.xls file (Dataset S1), where the *wbbYZ* Blast results are highlighted in green and the Kaptive results in orange.. To identify *wbbY* and w*bbZ* genes in other bacteria (Table S2), tBLASTn searches were performed using *wbbZ* as a query and the hits were manually searched for *wbbY*. The functions of any unknown genes in the vicinity of *wbbYZ* were predicted using BLAST, and the assignment of O serotypes for the *E. coli* isolates was based on comparison with published O-antigen clusters (19).

To generate a phylogenetic tree of polysaccharide pyruvyltransferases, puyruvate-containing bacterial glycans were selected from the Carbohydrate Structure Database (20) and (where available) the corresponding biosynthetic gene loci from genome data for the specific strains or serotypes were examined directly for genes encoding WbbZ orthologs (Table S3**)**. The corresponding genes were aligned using MAFFT 7.0 (strategy: G-INS-I, scoring matrix: BLOSUM62, gap penalty: 1.53, offset value: 0). A phylogenetic tree was generated using MAFFT (6) using the neighbour joining method with all gap-free sites and the Jones-Taylor-Thornton substitution model. A bootstrap of 100 iterations was performed. The tree was visualized using Phylo.io (21). Protein structural models were generated using AlphaFold2 using the ColabFold open-source platform (22). Structures were visualized in PyMol v2.5.4 (https://pymol.org/2/).

SI References

1. A. S. Shashkov, *et al.*, Full structure of the O-specific polysaccharide of *Proteus mirabilis* O24 containing 3,4-O-[(S)-1-carboxyethylidene]-D-galactose. *Carbohyd Res* 329, 453–7 (2000).

2. B. R. Clarke, *et al.*, A bifunctional O-antigen polymerase structure reveals a new glycosyltransferase family. *Nat Chem Biol* 16, 450–457 (2020).

3. S. D. Kelly, *et al.*, *Klebsiella pneumoniae* O1 and O2ac antigens provide prototypes for an unusual strategy for polysaccharide antigen diversification. *J Biol Chem* 294, 10863–10876 (2019).

4. B. R. Clarke, *et al.*, Molecular basis for the structural diversity in serogroup O2-antigen polysaccharides in *Klebsiella pneumoniae*. *J Biol Chem* 293, 4666–4679 (2018).

5. R. Köplin, J. R. Brisson, C. Whitfield, UDP-galactofuranose precursor required for formation of the lipopolysaccharide O antigen of *Klebsiella pneumoniae* serotype O1 is synthesized by the product of the $rfbD_{KPO1}$ gene. *J Biol Chem* 272, 4121–4128 (1997).

6. K. Katoh, J. Rozewicki, K. D. Yamada, MAFFT online service: multiple sequence alignment, interactive sequence choice and visualization. *Briefings Bioinf* 30, 3059 (2017).

7. P. Gouet, X. Robert, Deciphering key features in protein structures with the new ENDscript server. *Nucleic Acids Res* 42, W320–W324 (2014).

8. K. L. McCallum, G. Schoenhals, D. Laakso, B. Clarke, C. Whitfield, A high-molecular-weight fraction of smooth lipopolysaccharide in *Klebsiella* serotype O1:K20 contains a unique O-antigen epitope and determines resistance to nonspecific serum killing. *Infect Immun* 57, 3816–3822 (1989).

9. K. A. Datsenko, B. L. Wanner, One-step inactivation of chromosomal genes in Escherichia coli K-12 using PCR products. *Proc Natl Acad Sci USA* 97, 6640–6645 (2000).

10. R. G. Taylor, D. C. Walker, R. R. McInnes, *E. coli* host strains significantly affect the quality of small scale plasmid DNA preparations used for sequencing. *Nucleic Acids Res* 21, 1677–8 (1993).

11. M. F. Feldman, *et al.*, Engineering N-linked protein glycosylation with diverse O antigen lipopolysaccharide structures in *Escherichia coli*. *Proc Natl Acad Sci USA* 102, 3016–3021 (2005).

12. P. J. Hitchcock, T. M. Brown, Morphological heterogeneity among *Salmonella* lipopolysaccharide chemotypes in silver-stained polyacrylamide gels. *J Bacteriol* 154, 269–277 (1983).

13. M. A. Apicella, Isolation and characterization of lipopolysaccharides. *Meths Mol Biol* 431, 3–13 (2008).

14. Perdomo, Montero, Purification of *E. coli* 055:B5 lipopolysaccharides by size exclusion chromatography. *Biotecnol Aplic* 123, 124–129 (2006).

15. V. Kos, L. Cuthbertson, C. Whitfield, The *Klebsiella pneumoniae* O2a antigen defines a second mechanism for O antigen ATP-binding cassette transporters. *J Biol Chem* 284, 2947–2956 (2009).

16. L. M. Guzman, D. Belin, M. J. Carson, J. Beckwith, Tight regulation, modulation, and high-level expression by vectors containing the arabinose P_{BAD} promoter. *J Bacteriol* 177, 4121–4130 (1995).

17. F. Bolivar, *et al.*, Construction and characterization of new cloning vehicle. II. A multipurpose cloning system. *Gene* 2, 95–113 (1977).

18. S. David, *et al.*, Epidemic of carbapenem-resistant *Klebsiella pneumoniae* in Europe is driven by nosocomial spread. *Nat Microbiol* 4, 1919–1929 (2019).

19. B. Liu, *et al.*, Structure and genetics of *Escherichia coli* O antigens. *FEMS Microbiol Rev* 44, 655–683 (2020).

20. P. V. Toukach, K. S. Egorova, Carbohydrate structure database merged from bacterial, archaeal, plant and fungal parts. *Nucleic Acids Research* 44, D1229–D1236 (2016).

21. O. Robinson, D. Dylus, C. Dessimoz, Phylo.io: Interactive viewing and comparison of large phylogenetic trees on the web. *Mol Biol Evol* 33, 2163–2166 (2016).

22. M. Mirdita, *et al.*, ColabFold: making protein folding accessible to all. *Nat Methods* 19, 679–682 (2022).
